# Shipped and shifted: modeling collection-induced bias in microbiome multi-omics using a tractable fermentation system

**DOI:** 10.1038/s41522-025-00909-1

**Published:** 2026-01-09

**Authors:** Annina R. Meyer, Jan Patrick Tan, Mihnea Paul Mihaila, Michelle Neugebauer, Laura Nyström, Nicholas A. Bokulich

**Affiliations:** 1 Laboratory of Food Systems Biotechnology, Department of Health Sciences and Technology, ETH Zurich, Zurich, Switzerland; 2 Laboratory of Food Biochemistry, Department of Health Sciences and Technology, ETH Zurich, Zurich, Switzerland

**Keywords:** Computational biology and bioinformatics, Ecology, Ecology, Microbiology, Systems biology

## Abstract

Large-scale, decentralized microbiome sampling surveys and citizen science initiatives often require periods of storage at ambient temperature, potentially altering sample composition during collection and transport. We developed a generalizable framework to quantify and model these biases using sourdough as a tractable fermentation system, with samples subjected to controlled storage conditions (4 °C, 17 °C, 30 °C, regularly sampled up to 28 days). Machine-learning models paired with multi-omics profiling—including microbiome, targeted and untargeted metabolome profiling, and cultivation—revealed temperature-dependent shifts in bacterial community structure and metabolic profiles, while fungal communities remained stable. Storage induced ecological restructuring, marked by reduced network modularity and increased centrality of dominant taxa at higher temperatures. Notably, storage duration and temperature were strongly encoded in the multi-omics data, with temperature exerting a more pronounced influence than time. 24 of the top 25 predictors of storage condition were metabolites, underscoring functional layers as both sensitive to and informative of environmental exposure. These findings demonstrate that even short-term ambient storage (<2 days) can substantially reshape microbiome, metabolome, and biochemical profiles, posing risks to data comparability in decentralized studies and emphasizing the need to recognize and address such biases. Critically, the high predictability of storage history offers a path toward bias detection and correction— particularly when standardized collection protocols are infeasible, as is common in decentralized sampling contexts. Our approach enables robust quantification and modeling of such storage effects across multi-omics datasets, unlocking more accurate interpretation of large-scale microbiome surveys.

## Introduction

Complex microbial ecosystems—microbiomes—colonize nearly every corner of our planet, playing key roles in food production as well as human, animal, plant, and planetary health^1–3^. Accurate characterization of microbiomes and metabolome composition and function critically depends on standardized protocols for sample collection, storage, and handling. Even minor deviations in storage conditions can shift microbial community structure, altering metabolic outputs. These shifts can bias downstream analyses and compromise data comparability, as demonstrated in aquatic, soil, and gut microbiomes^4–8^. While immediate freezing at −20 °C or −80 °C remains the gold standard to minimize microbial distortion in fecal microbiomes, as confirmed by both amplicon^9,10^ and whole metagenome sequencing^11^, it is often infeasible in decentralized, large-scale sampling efforts and in field studies. To limit compositional drift during room-temperature exposure, alternative stabilization strategies (e.g., sampling kits, storage conditions) have been benchmarked specifically for fecal samples^12,13^. However, these tests were matrix-specific and do not necessarily generalize to diverse biological or food microbiomes, which ideally both need to be biobanked and preserved for sequencing-based characterization.

This challenge is especially pronounced in citizen science and global-scale microbiome surveys, where samples are often shipped under heterogeneous ambient conditions without cold-chain logistics. In citizen science projects like the American Gut Project^6^, Global Sourdough Project^14^, and HealthFerm Project^15^, samples frequently experienced uncontrolled temperature exposure before processing and analysis in the laboratory. Although storage effects on individual omics layers—such as microbial community composition—have been investigated, and correction strategies have been proposed for fecal samples^6,16^, the extent and nature of such distortions across integrated multi-omics data and in non-fecal matrices such as food fermentations remain underexplored.

In fermented foods, residual substrates sustain metabolism, enabling not only the outgrowth of individual taxa but also community-wide restructuring in response to environmental stressors^17,18^. Refrigeration slows, but does not halt these dynamics, and for example psychrotrophic bacteria such as *Hafnia alvei* and *Pseudomonas* spp. remain metabolically highly active even at 4 °C^19^. Warmer storage accelerates metabolism and acidification, which initially contributes to food safety and spoilage prevention^20,21^. However, this can also accelerate community restructuring—particularly through the loss of lactic acid bacteria (LAB) viability^19,22–24^, favoring more acid- and heat-tolerant taxa^20^. These shifts undermine both microbiome stability and also functional attributes of the fermentation ecosystems.

In parallel to taxonomic shifts, prolonged storage can induce changes in the metabolite landscape. Residual sugars and organic acids present in fresh fermentations are transformed by ongoing microbial metabolism, and can further reshape the biochemical fingerprint of a ferment before visible changes in microbial composition occur^25^. Such transformations can obscure biological signals, compromise reproducibility, and confound interpretation in multi-omics studies.

Sourdough provides a tractable, ecologically relevant model to investigate these dynamics. Its microbiome, dominated by LAB, acetic acid bacteria, and yeasts, is highly responsive to environmental perturbations^14,26–29^. Moreover, compared with highly diverse host-associated and environmental microbiomes such as gut or soil communities, sourdough starters are typically dominated by a limited number of bacterial and fungal taxa, making them a tractable yet ecologically interesting model for mechanistic studies of community and metabolic resilience.

While fermentation conditions have been extensively studied^30–33^, the impact of post-sampling storage on sourdough microbiome and metabolome integrity remains poorly defined. Addressing this gap is critical—not only to safeguard interpretability in decentralized multi-omics studies of fermentations, but also to understand how environmental stress can reshape fermentation ecosystems, with potential implications for food quality and safety^34,35^.

**Fig. 1:**
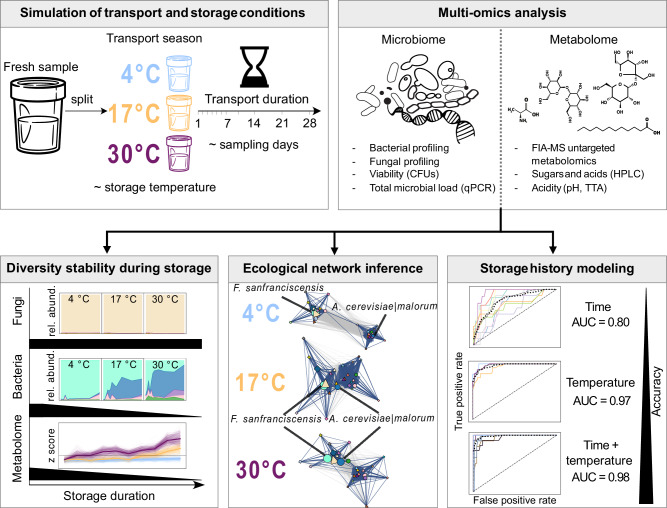
Overview of experimental design, multi-omics analysis, and main findings. A fresh sourdough starter was split and subjected to simulated post-collection storage scenarios across three temperatures (4 °C, 17 °C, and 30 °C), reflecting transport season. Samples were analyzed over 11 timepoints spanning 28 days, representative for variable transport durations (day 1–7, 10, 14, 21 and 28). A multi-omic approach was applied, combining microbiome (16S rRNA v4/ITS amplicon sequencing, qPCR, colony forming units (CFUs)) and metabolome (flow injection analysis mass spectrometry (FIA-MS), high-pressure liquid chromatography (HPLC), acidity) analysis to assess microbial and functional stability after variable collection conditions. Analyses revealed that bacterial diversity and metabolite profiles diverged significantly with prolonged warm storage, while fungal communities remained comparatively stable. Network inference highlighted ecological restructuring, notably the rise in centrality of the acetic acid bacterium *Acetobacter cerevisiae|malorum* with increasing temperature. Finally, supervised machine learning showed that integrated multi-omic features are sensitive predictors of storage time and temperature (AUC = 0.98), offering a basis for future bias detection and potential correction in decentralized microbiome studies.

Here, we used sourdough as a tractable model fermentation system to investigate how realistic post-sampling storage conditions affect microbiome and metabolome profiles (Fig. 1). To simulate common sample shipping scenarios, sourdough aliquots were incubated at 4 °C, 17 °C, or 30 °C for up to 28 days without substrate replenishment. A temporal multi-omics design—spanning 16S rRNA and ITS amplicon sequencing, cultivation, untargeted FIA-MS metabolomics, and HPLC profiling—was used to dissect the progression of microbial and biochemical changes across storage conditions. Using machine learning, we demonstrated that both storage time and temperature were strongly and predictably encoded in multi-omics profiles, revealing consistent microbial and metabolic signatures of post-sampling environmental exposure. These patterns underscore the need for caution when interpreting data from decentralized sampling efforts, where storage conditions may confound biological signals.

## Results

### Temperature and time drive microbial viability loss and biochemical divergence in sourdough starters during simulated transport

Large-scale microbiome initiatives such as citizen science projects expand access to diverse ecosystems but often rely on ambient-temperature collection and transport, raising concerns about post-collection storage-induced bias. To quantify these effects under a controlled experimental setting, we simulated typical transport conditions using sourdough aliquots incubated at 4 °C, 17 °C, or 30 °C—representing seasonal ambient temperatures—over 28 days without substrate replenishment (Fig. 1).

**Fig. 2:**
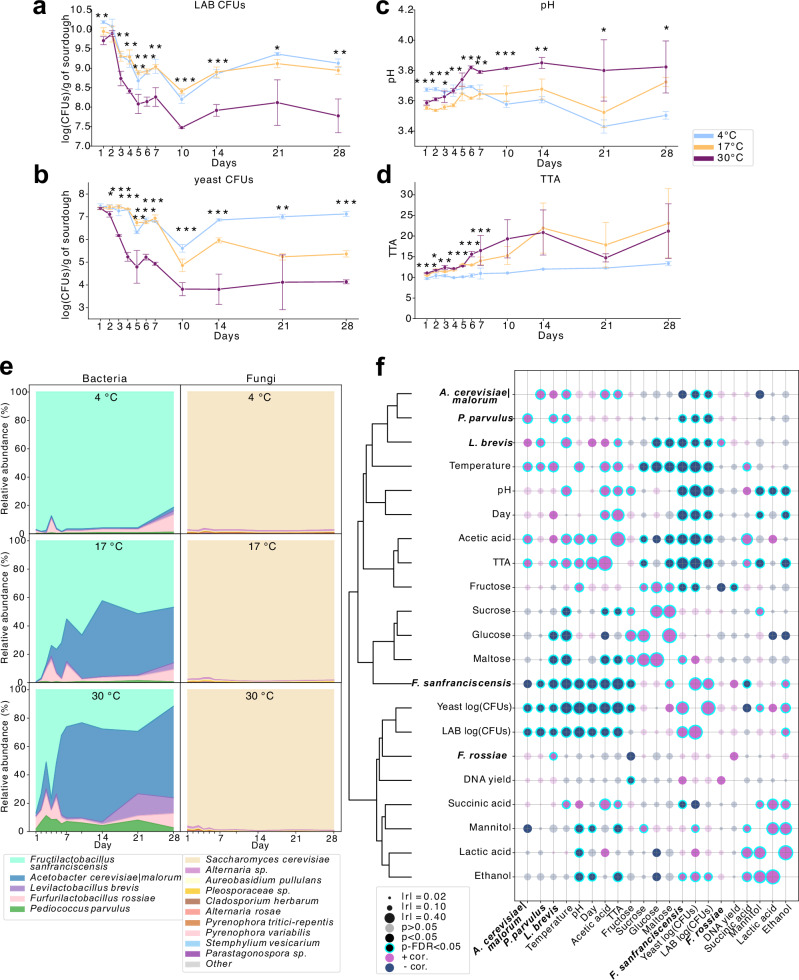
Simulated large-scale sourdough sampling reveals significant temperature- and time-dependent shifts in microbial and biochemical profiles. **a**–**d** Temporal dynamics of lactic acid bacteria (LAB) CFUs, yeast CFUs, pH, and total titratable acidity (TTA). Statistical differences across temperatures at each time point were assessed by one-way ANOVA with FDR correction (*p < 0.05, **p < 0.01, ***p < 0.001). **e** Relative abundance of bacterial (16S rRNA) and fungal (ITS) communities across storage conditions. Fungal taxa <0.05% were grouped as “Other”. **f** Pearson correlation matrix of physicochemical and molecular parameters, including pH, TTA, CFUs, DNA yields, qPCR-based bacterial load, and HPLC-derived sugars and acids. Features were hierarchically clustered by pairwise correlations (r-values). All data represent biological triplicates.

At each time point, three biological replicates per temperature condition were analyzed. Microbiological analyses revealed rapid declines in viable lactic acid bacteria (LAB) and yeast at higher temperatures. By day 1, LAB colony forming units (CFUs) were already reduced at 30°C relative to cooler conditions (Fig. 2a), with significant within-group declines from day 3 onward. Yeast viability declined from day 4–5 (Fig. 2b, Fig. S2c, d). For reference, measurements of sourdough characteristics (microbial and chemical) at day 0 are shown in the supplementary figures (e.g., Fig. S1), but were not included in the statistical comparisons because day 0 corresponds to the single mother sourdough batch prior to exposure to the experimental storage conditions (see Methods for details).

Storage temperature also impacted acidity and metabolite dynamics. pH declined gradually at 4 °C but increased over time at 17 °C and 30 °C (Fig. 2c, Fig. S2a), while total titratable acidity (TTA) rose across all conditions, with a sharper increase at elevated temperatures—doubling by day 14 (Fig. 2d, Fig. S2b). These shifts reflect accelerated microbial metabolism under non-cooled conditions, further evidenced by high-pressure liquid chromatography (HPLC)-based metabolite profiling (Figs. S1–S3). Acetic acid showed accumulation at 17 °C and 30 °C from day six onwards (Fig. S1e), whereas lactic acid, succinic acid, and ethanol remained comparatively stable (Figs. S1f–h, S2e, f, S3a, b). Sugars such as glucose, maltose, and sucrose were rapidly depleted at warmer temperatures (Fig. S1i, k, l), followed by glucose reappearance from day 5–7 onward at 30 °C (Fig. S3c). Fructose accumulation from day 14 at 30 °C likely reflects enhanced enzymatic hydrolysis of oligosaccharides at elevated temperatures, potentially coupled with reduced microbial uptake or conversion (Fig. S1j). The concurrent stability of mannitol levels across cold conditions, and slight decrease at elevated temperatures (Fig. S1m, S3g) may suggest a temperature-dependent suppression of fructose-to-mannitol conversion, which may be linked to the activity or abundance of mannitol-producing heterofermentative LAB. We also observed substantial between-replicate variability in absolute acidity measurements, sugar or acid concentrations at each time point (Figs. S2, S3). This variability reflects heterogeneity among independent semi-solid fermentations in separate containers, rather than technical noise or oscillations within a single dough, and is consistent with the inherent microscale heterogeneity of sourdough matrices.

### Higher temperature catalyzes microbial succession and promotes starvation tolerant taxa to bloom

Microbial community trajectories were profiled using 16S rRNA (V4) and ITS amplicon sequencing. The baseline bacterial community consisted of five species — *Fructilactobacillus sanfranciscensis*, *Levilactobacillus brevis*, *Furfurilactobacillus rossiae*, *Pediococcus parvulus, and Acetobacter cerevisiae|Acetobacter malorum* (indistinguishable 16S v4 and the type strains share 99.9% full-length 16S rRNA gene similarity^36^; hereafter we refer to this ASV assignment as *A. cerevisiae|malorum). Saccharomyces cerevisiae*, out of 27 detected fungal species, dominated the fungal community across all samples (>97.5% relative abundance; Fig. 2e). At 4 °C, bacterial relative abundances remained stable throughout the one month period under starvation. However, warm conditions favored rapid expansion of *A. cerevisiae|malorum* (at 17 °C from day 3, and 30 °C from day 1), and also supported increased abundance of *L. brevis* and *P. parvulus*, while *F. sanfranciscensis* declined with increasing storage temperature and time (Fig. 2e).

To validate these trends and account for compositional bias, total bacterial load was quantified via 16S rRNA qPCR (Fig. S4a, d). Neither total abundance nor DNA yield varied significantly across time or temperature (Fig. S4b, c). Combining qPCR with relative abundance data enabled absolute quantification of species dynamics: *F. sanfranciscensis* remained dominant at 4 °C; at 17 °C, *A. cerevisiae|malorum* overtook by day 14; and at 30 °C, *A. cerevisiae|malorum* dominated by day 6 (Fig. S4e).

Correlational clustering of microbial, physicochemical, and metabolite data contextualized these shifts (Fig. 2f). *A. cerevisiae|malorum*, *P. parvulus*, and *L. brevis* co-clustered with temperature and pH, linking their activity to warming and acidification. This group was closely aligned with acetic acid, TTA, and fructose, indicating that elevated TTA is primarily driven by higher acetic acid accumulation relative to lactic acid. This is consistent with the higher pKₐ (≈4.75) of acetic acid compared to lactic acid (pKₐ ≈ 3.86). Conversely, *F. sanfranciscensis* clustered with readily fermentable sugars (glucose, maltose, sucrose), highlighting its more strict dependency on primary carbohydrate availability^37^. LAB and yeast CFUs, DNA yields, and residual sugars and acids formed a final cluster reflecting shared responses to broader ecological restructuring (Fig. 2f).

### Compositional restructuring hints at competitive dynamics under varying storage conditions

**Fig. 3:**
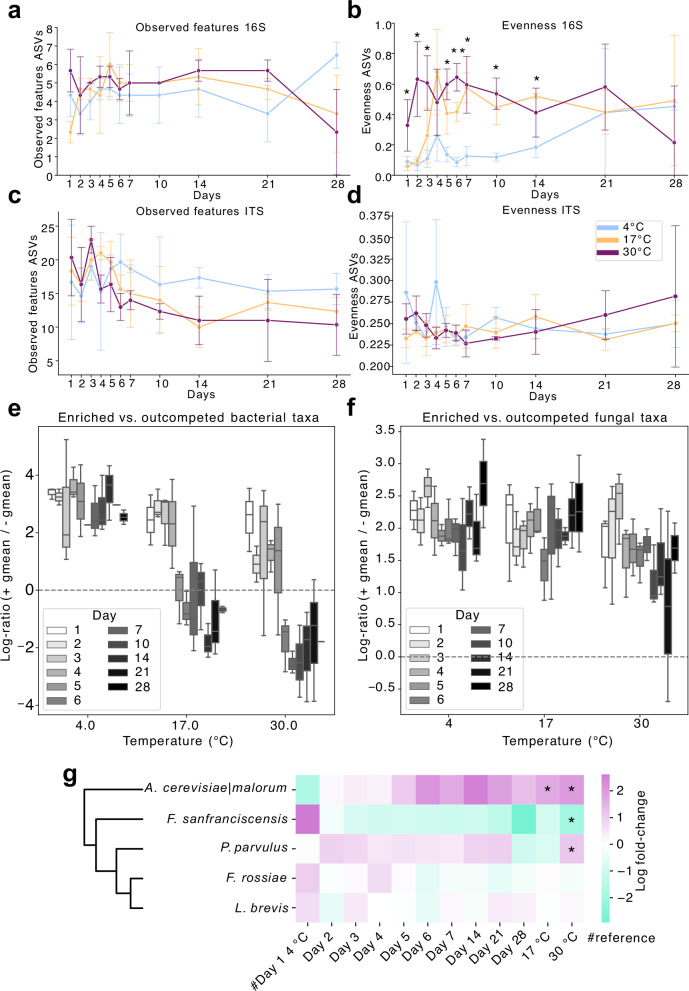
Storage temperature and duration drive bacterial, but not fungal, community restructuring in sourdough. Alpha-diversity of bacterial (16S; **a**, **b**) and fungal (ITS; **c**, **d**) communities over time and across storage temperatures. Richness (observed features) and evenness were calculated from ASV-level data; significance is assessed via one-way ANOVA with FDR correction (*p < 0.05, **p < 0.01, ***p < .001). **e**, **f** Log-ratios of enriched versus outcompeted taxa based on reference group enrichments, with the reference for all comparisons being day 1 at 4 °C (ANCOM-BC2, Table S5 and S6), highlight pronounced bacterial compositional turnover at 17 °C and 30 °C, absent at 4 °C and across fungal communities. **g** Differential abundance heatmap of bacterial taxa across conditions. Log-fold changes (relative to day 1 at 4 °C, again serving as reference for all comparisons across all temperatures and time points) were computed via ANCOM-BC2; taxa hierarchically clustered using average linkage (Euclidean distance). Asterisks indicate significant differential abundances (FDR-adjusted q < 0.05).

Alpha-diversity analyses reinforced the temperature- and time-driven bacterial succession observed in abundance profiles. Richness did not significantly change across any conditions for both bacteria and fungi, regardless of taxonomic resolution (ASV, OTU, k-mer; Fig. 3a, c; Fig. S5a, b, d). However, bacterial evenness increased significantly—by day 1 at 30 °C and from day 4 at 17 °C—indicating selective expansion of certain taxa under warmer conditions (Fig. 3b). Fungal evenness remained unchanged (Fig. 3d), consistent with *S. cerevisiae*’s near-total dominance across samples.

To capture compositional imbalance, we applied ANCOM-BC2 differential abundance modeling. This revealed sharp declines in the log-ratio of enriched to outcompeted bacterial taxa between days 4–5 at 17 °C and days 5–6 at 30 °C (Fig. 3e), reflecting a community-wide turnover. No such restructuring occurred at 4 °C or in fungal communities at any temperature (Fig. 3f; Fig. S6), highlighting bacterial communities’ higher sensitivity to storage induced metabolic stress in this model system. Strikingly, *A. cerevisiae|malorum* and *F. sanfranciscensis* exhibited strongly opposing trajectories across all timepoints and temperatures (Fig. 3g), supporting a model of contrasting ecological adaptations: *F. sanfranciscensis* thrives in cool, sugar-rich conditions, whereas *A. cerevisiae|malorum* is better adapted to warm, more nutrient-depleted and acidified environments.

### Temperature and time shape bacterial and fungal beta-diversity in opposite directions

**Fig. 4:**
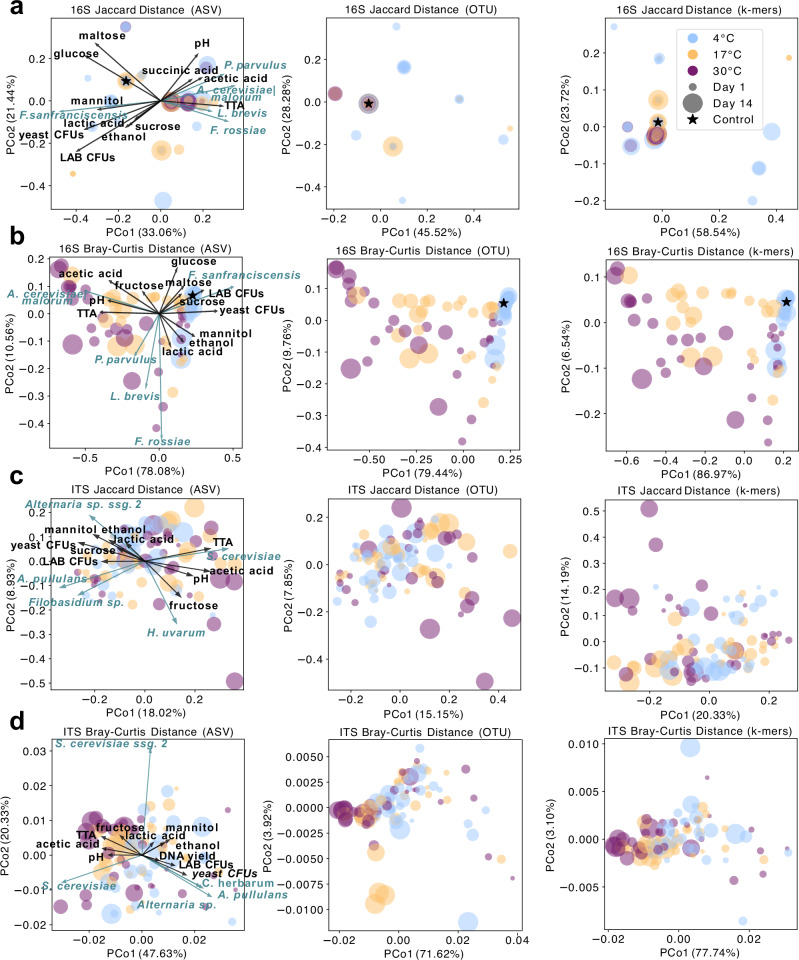
Storage modulates microbial community structure via compositional shifts, captured most sensitively by k-mer-based profiling. PCoA ordinations of bacterial (16S; **a**, **b**) and fungal (ITS; **c**, **d**) communities based on Jaccard (**a**, **c**) and Bray-Curtis (**b**, **d**) distance metrics across ASV, OTU, and k-mer resolutions. For ASV-based ordinations, the top taxa and metadata most correlated with the first two PCoA axes are overlaid as arrows, with direction and length reflecting Pearson’s r values. Arrows are scaled by the sum of correlations with PCo1 and PCo2. The ‘control’ refers to the mother sourdough at day 0 before splitting and incubating sourdoughs at either 4 °C, 17 °C or 30 °C; no ITS control is shown due to rarefaction-driven sample loss.

To dissect microbial restructuring under storage, we analyzed beta-diversity in bacterial (Fig. 4a, b) and fungal (Fig. 4c, d) communities. Among bacteria, Bray–Curtis distances captured greater variance than Jaccard across all feature levels, with k-mer-based PCoA explaining the most (PCo1: 86.97%, PCo2: 6.54%), outperforming OTUs (79.44%/9.76%) and ASVs (78.0%/10.56%). This highlights the superior resolution of k-mers, even within a low-complexity system like sourdough.

Biplot overlays revealed that 4 °C samples clustered with higher glucose, maltose, sucrose, mannitol, ethanol, lactic acid, CFU counts, and *F. sanfranciscensis*, while 17 °C and 30 °C samples correlated with *A. cerevisiae|malorum*, elevated acetic acid levels, TTA, pH, and fructose (Fig. 4b).

Fungal beta-diversity was less temperature-dependent, though Bray–Curtis again explained more variance. Two *S. cerevisiae* ASVs associated with high temperature and long storage correlated with TTA, pH, acetic acid, and fructose, while 4 °C samples were enriched in minor taxa, DNA yield, CFUs, mannitol, and ethanol (Fig. 4d).

Beta-dispersion analyses revealed rising bacterial heterogeneity with storage duration and temperature, as shown by increasing mean distances to centroid: 0.049 (4 °C), 0.240 (17 °C), 0.337 (30 °C), and over time from 0.064 (day 1) to 0.218 (day 4) and 0.356 (day 21). PERMDISP confirmed significant dispersion increases (temperature: F_ASV = 40.262, F_OTU = 46.166, F_kmer = 42.082; day: F_ASV = 3.508, F_OTU = 3.413, F_kmer = 4.160; all p = 0.001). In contrast, fungal dispersion remained stable (mean distance to centroid: 0.016–0.018; PERMDISP p > 0.2), indicating higher resilience to storage-induced stress.

PERMANOVA supported these patterns: for bacteria, variance was primarily explained by temperature (R² = 0.36, p = 0.001), followed by nested effects (temperature(day), R² = 0.35, p = 0.001), and day (R² = 0.14, p = 0.001) (Fig. S7a); for fungi, day had stronger effects (R² = 0.14, p = 0.001) than temperature (R² = 0.04, p = 0.009) (Fig. S7d). Nesting by temperature within day explained more variance than the inverse in both kingdoms (Fig. S7c, f).

Notably, nested PERMANOVA revealed divergent temperature-dependence: bacterial communities showed greatest temporal variance at 4 °C (R² = 5.168, p = 0.006), decreasing at 17 °C (R² = 4.284, p = 0.001) and 30 °C (R² = 3.164, p = 0.006); fungi displayed the opposite trend, with variance increasing from 4 °C (R² = 1.155, p = 0.320) to 30 °C (R² = 2.632, p = 0.007). All together, this suggests that while bacterial communities at higher temperatures became more compositionally variable, these changes were also more stochastic across replicates. Fungal communities, by contrast, exhibited stable dispersion but increasing PERMANOVA R² at higher temperatures, pointing to greater susceptibility to temporal restructuring as compared to bacterial communities, which was consistently shared across samples.

### Untargeted metabolomics reveals temperature- and time-dependent metabolite clusters with distinct functional enrichments

To expand beyond targeted chemical analysis, we applied untargeted FIA-MS metabolomics, detecting 1097 ion features across all sourdough samples (Fig. S8). Elevated temperatures led to increased metabolic diversity: at 17 °C, the number of features doubled by day 7, and at 30 °C, this increase was already evident by day 2 compared to 4 °C and day 1 (Fig. S8a, c). In line with bacterial patterns, feature evenness remained stable at 4 °C but rose progressively at 17 °C and more rapidly at 30 °C (Fig. S8b, d).

**Fig. 5:**
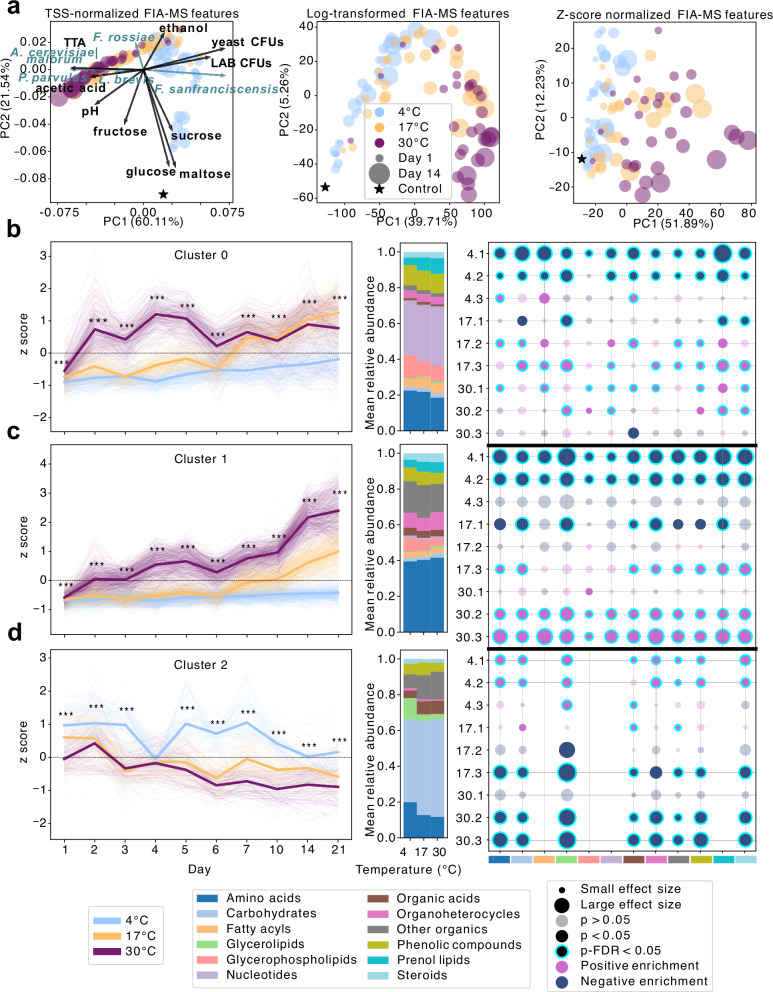
Storage conditions restructure the sourdough metabolome. **a** Principal component analysis (PCA) of FIA-MS metabolite profiles under varying normalization and transformation strategies demonstrates how data preprocessing influences separation by temperature and explained variance. In the TSS-normalized ordination (left), key bacterial taxa and selected HPLC/metadata variables were projected as arrows. Arrow direction and length reflect Pearson correlations with PC1 and PC2, scaled to the sum of absolute correlations. Temperature- and time-responsive metabolites were clustered via k-means into three dynamic groups: Cluster 0 (**b**), Cluster 1 (**c**), and Cluster 2 (**d**). Z-scored metabolite trajectories are shown per temperature, with fine lines for individual features and bold lines for group means. Temperature effects at each time point were assessed via Kruskal–Wallis tests with FDR correction (*p < 0.05, **p < 0.01, ***p < 0.001). Bar plots show the average class-level metabolite abundances across temperature conditions. To identify associations between metabolite classes, time, and temperature, Mann–Whitney U tests were performed by comparing each feature in a given condition against all others. Time was binned into three phases: Phase 1 (days 1–4), Phase 2 (days 5–10), and Phase 3 (days 14–21). Each time phase was further stratified by temperature, with condition labels such as, e.g., 4.1 (Phase 1 at 4 °C) or 17.1 (Phase 1 at 17 °C). Enrichment was calculated as the log fold-change between condition-specific and global means. Statistically enriched metabolite classes (after BH-FDR correction) are annotated.

Principal component analysis of FIA-MS profiles under different normalization schemes (TSS, log, z-score) revealed temperature-driven clustering analogous to 16S Bray–Curtis ordinations (Fig. 5a). TSS normalization provided the clearest separation (PC1 = 60.11%, PC2 = 21.54%), outperforming log (39.71%/5.26%) and z-score (51.89%/12.23%). Bi-plot overlays indicated strong correlations between 4 °C samples and fermentable sugars (sucrose, glucose, maltose), *F. sanfranciscensis*, CFUs, and ethanol. In contrast, warmer storage aligned with increased concentrations of acetic acid, pH, TTA, *A. cerevisiae|malorum*, and subdominant taxa (*P. parvulus, L. brevis, F. rossiae*) (Fig. 5a).

Procrustes analysis demonstrated strong concordance between metabolomic and bacterial community structures: FIA_TSS vs. 16S PCoA (M² = 0.437, p = 0.001), FIA_TSS PCA vs. 16S PCoA (M² = 0.498, p = 0.001), and FIA z-score vs. 16S (M² = 0.591, p = 0.001), with TSS offering the best alignment (Table S7). Concordance was highest at 17 °C (M² = 0.438), followed by 30 °C (M² = 0.593), and was non-significant at 4 °C (M² = 0.987, p = 0.718). Temporally, microbiome–metabolome synchrony peaked between days 6–14 (M² = 0.241–0.277, p < 0.01), diminishing during early and late storage phases (Table S8).

K-means clustering partitioned the FIA-MS features into three distinct temporal profiles (Fig. 5b–d), each reflecting coordinated biochemical responses (Kyoto Encyclopedia of Genes and Genomes (KEGG) + (Human Metabolome Database) HMD annotation ±0.002 Da, categorization into fermentation-relevant metabolite classes following the HMDB metabolite taxonomy).

- Cluster 0: Nucleotides, aromatic compounds, hormones, and toxin-like metabolites remained stable at 4 °C but increased at 17 °C and 30 °C (Fig. 5b), likely reflecting a combination of enhanced nucleotide turnover and passive release via cellular lysis under nutrient limitation and increased (auto-)acidification at elevated temperatures. Similar shifts in nucleotide pool shifts have been reported in lactic acid bacteria under nutrient limitation, acid, and oxidative stress which all stimulate increased nucleotide salvage, DNA/RNA repair, and the production of stress-signaling nucleotides such as c-di-AMP^38,39^.
- Cluster 1: Enriched in amino acids, fatty acyls, glycerolipids, organoheterocycles, prenol lipids, and steroids. These catabolic metabolites surged at 30 °C during days 5–10, appeared later at 17 °C, and remained depleted at 4 °C (Fig. 5c). These dynamics align with pronounced proteolysis, lipid remodeling, and stringent response mediated amino acid recycling under starvation stress and elevated temperatures^39^. However, given the absence of significant total bacterial biomass accumulation (qPCR, Fig. S4e), non-microbial sources such as endogenous wheat proteases and lipoxygenases may also contribute, particularly in response to thermal activation.
- Cluster 2: Carbohydrate-related features (oligosaccharides, sugar alcohols) showed depletion at elevated temperatures and stability or slight enrichment in early phases at 4 °C (Fig. 5d), mirroring faster sugar consumption due to elevated enzymatic activity at warmer storage conditions with progressing time.

Together, these trajectories reflect a temperature-dependent modulation of metabolic activity, with carbohydrate catabolism occurring more rapidly from day 1 onwards at warmer temperatures, and more gradually at 4 °C. The subsequent accumulation of proteolytic and nucleotide-associated metabolites at 17 °C and 30 °C, and their delayed rise at 4 °C (e.g., Cluster 0), suggest that core metabolic processes proceed at different rates depending on storage temperature. Overall, the data support a model of progressive, rate-controlled metabolic layering, in which warmer conditions accelerate both the onset and magnitude of nutrient stress responses such as proteolysis and nucleotide turnover.

### Storage temperature restructures microbial-metabolite networks

**Fig. 6:**
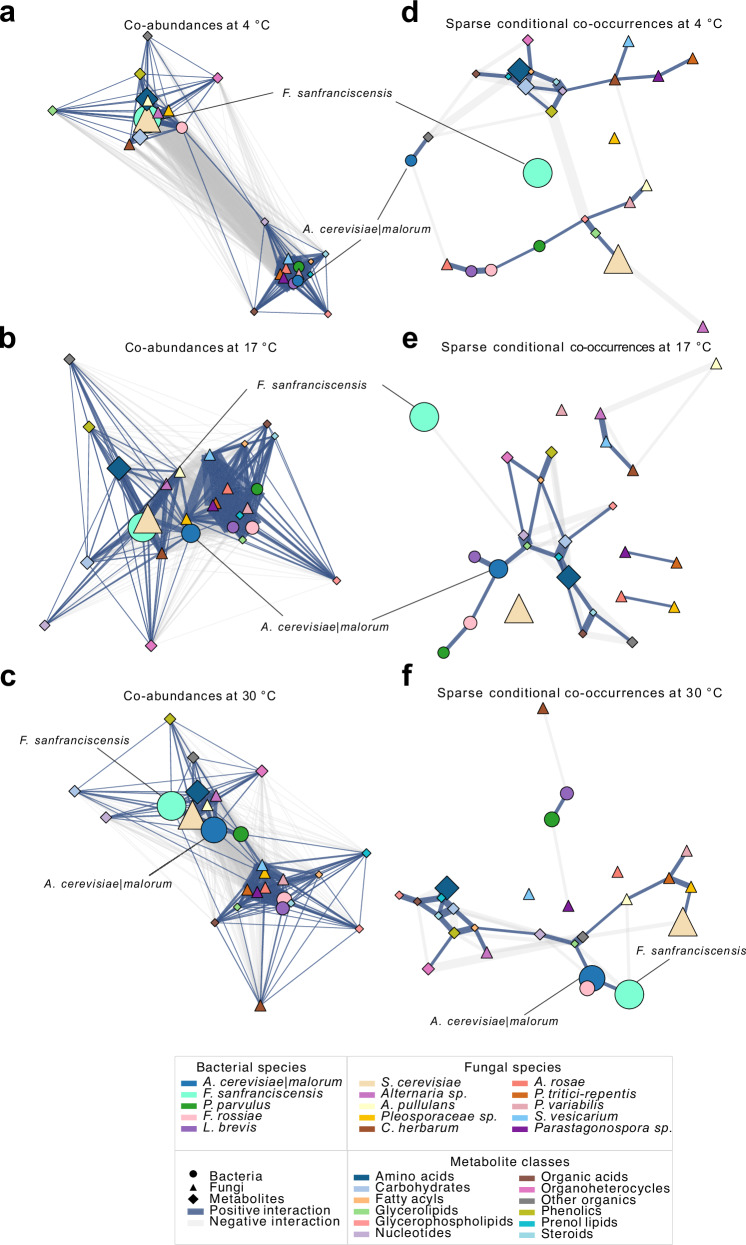
Ecological networks in sourdough microbiomes under starvation at different temperatures. **a**–**c** Abundance weighted co‑occurrence networks (CLR-transformed) reveal temperature-dependent microbial–metabolite association modules. At 4 °C, *F. sanfranciscensis* and yeast taxa dominate, while at 17 °C and especially 30 °C, *A. cerevisiae|malorum* becomes central, bridging core and subdominant taxa. **d**–**f** Sparse inverse-covariance networks capture conditional dependencies: at low temperature, co-occurrences are compartmentalized; at higher temperatures, *A. cerevisiae|malorum* is a clear keystone, showing direct conditional links to both bacterial and metabolite nodes.

To investigate how storage conditions reshape ecosystem connectivity, we constructed abundance-weighted co-correlation networks (Fig. 6a–c) and sparse conditional co-occurrence networks (Fig. 6d–f) for each storage temperature (4 °C, 17 °C, 30 °C). In the co-correlation networks, two major modules consistently emerged: the one containing dominant taxa and their metabolite partners, and the second one harboring subdominant species. At 4 °C, *F. sanfranciscensis, F. rossiae*, and *S. cerevisiae* dominated the core cluster (Fig. 6a). At 17 °C, network modularity diminished as clusters became more interconnected, with overall stronger co-correlations (Fig. 6b). By 30 °C, *A. cerevisiae|malorum* transitioned from the subdominant cluster (4 °C) to the inter-module bridge (17 °C) and ultimately joined the dominant hub (Fig. 6c). This trajectory was mirrored by increasing betweenness and eigenvector centrality for *A. cerevisiae|malorum*, and a concurrent decline in centrality for *F. sanfranciscensis* (Fig. S9).

Sparse conditional networks uncovered temperature-specific rewiring of direct associations. At 4 °C, *F. sanfranciscensis* remained largely disconnected, with no significant positive microbiome or metabolome associations, while less dominant bacterial species exhibited stronger connectivity (Fig. 6d). Centrality analyses confirmed *F. sanfranciscensis* had the lowest centrality values at 4 °C (Fig. S10), whereas *A. cerevisiae|malorum* retained higher influence across all temperatures. At 30 °C, *F. sanfranciscensis* gained closeness centrality and established a positive edge with *A. cerevisiae|malorum* (Fig. 6f), suggesting increased ecological interdependence in the bacterial sourdough community under warmer fermentation conditions.

Together, these network analyses reveal that rising temperatures not only shift microbial abundances but also rewire co-occurrence topology—amplifying the integrative role of *A. cerevisiae|malorum* while diminishing the dominance of *F. sanfranciscensis*. This rewiring may reflect broader ecological adaptation across microbial and metabolic layers.

### Functional metabolite profiles outperform taxonomic composition in predicting storage history

**Fig. 7:**
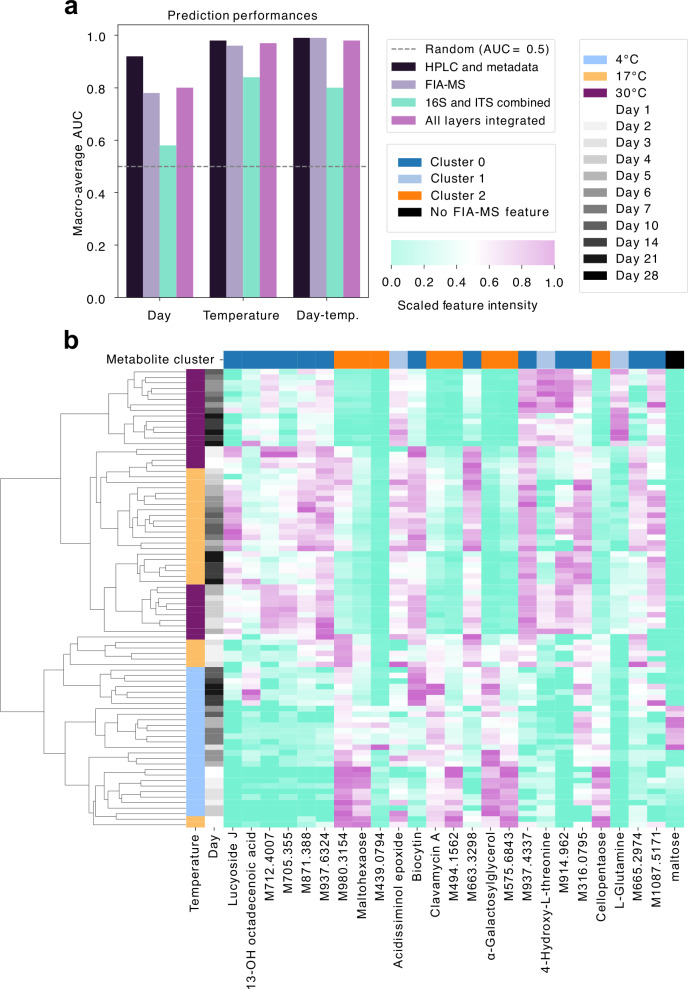
Storage-phase classification and predictive markers in sourdough microbiomes. **a** Accuracy of the predictive performance for storage day, temperature, and combined day–temperature categories based on different data types: HPLC and metadata (including CFU counts, pH, and TTA), full FIA–MS metabolome profiles (annotated and unannotated), combined 16S and ITS microbiome data, and the fully integrated multi-omics dataset. Predictions were generated using cross-validated random forest (RF) models. **b** Top 25 predictive features: strongest signals derive from metabolomics (Clusters 0–2), complemented by HPLC‑measured sugars and acids and CFUs, the heatmap shows hierarchical clustering by storage phase (time x temperature) across metabolite clusters.

To identify which features most robustly reflect storage-induced perturbations, we benchmarked predictive performance of individual and integrated data layers—including amplicon-based microbiome profiles, untargeted FIA-MS metabolomics, and physicochemical metadata (HPLC sugars/acids, CFUs, pH, TTA)—using cross-validated random forest models (Fig. 7a, Fig. S11).

Storage duration was most accurately predicted from HPLC and metadata (macro-average AUC = 0.92), with LAB CFUs, fructose, and mannitol as top-ranked features (Fig. S12a). Predictive performance for storage duration based on the microbiome was poor (AUC = 0.58), and driven by low-abundance fungi (e.g., *P. variabilis*, *D. fristingensis*, *A. pullulans*) and *A. cerevisiae|malorum* (Fig. S13a), highlighting limited temporal specificity in community composition alone. Storage temperature was again best captured by HPLC features and metadata (AUC = 0.98), dominated by maltose, glucose, and TTA (Fig. S12b). The microbiome was more predictive for temperature (AUC = 0.84) than time, where most discriminatory features included *F. sanfranciscensis*, *P. parvulus*, and *P. variabilis* (Fig. S13b). This likely reflects their differential resilience to temperature-induced accelerated acidification, contributing to temperature-dependent niche structuring and community dynamics^27,39^.

Classification of combined temperature–time phases (Phase 1: days 1–4, Phase 2: days 5–10, Phase 3: days 14–28) achieved near-perfect accuracy with FIA-MS (AUC = 0.99), HPLC + metadata (AUC = 0.99), and full multi-omics integration (AUC = 0.98). Using microbiome data alone demonstrated high accuracy but inferior performance (AUC = 0.80) (Fig. S11). Strikingly, 24 of the top 25 predictive features originated from the metabolome, particularly FIA-MS Cluster 0, enriched in nucleotides, steroids, and stress-response metabolites with increasing abundance under warmer conditions and extended storage (Fig. 7b, Fig. S13f). Additional contributions came from Clusters 1 (amino acids) and 2 (carbohydrates), reflecting metabolic transitions from carbohydrate depletion to proteolysis and secondary metabolite accumulation. HPLC-measured maltose was the only non-FIA-MS predictor in the top 25.

These results highlight that metabolite profiles—reflecting the functional output of microbial activity—more robustly capture storage history than taxonomic composition alone. This decoupling of function from community structure underscores the central role of metabolic adaptation in driving fermentation dynamics, particularly under varying temperature conditions and extended storage durations.

## Discussion

Decentralized microbiome sampling—crucial to citizen science and global biodiversity surveys—faces a persistent challenge: preserving data integrity in the absence of controlled post-collection storage. Understanding how environmental exposures can reshape microbial composition and function is therefore critical for elucidating ecological dynamics and eliminating bias in large-scale microbiome studies. Our multi-omics analysis revealed that even short-term ambient storage (17–30 °C) restructures microbial ecosystems across multiple omics layers. In a controlled sourdough model, bacterial communities, metabolite profiles, and ecological network topology underwent pronounced shifts with storage temperature and duration—while fungal communities remained stable. These results highlight the higher sensitivity of bacterial and functional layers to environmental perturbation as compared to the fungal community, as observed in our relatively low-complexity, closed sourdough ecosystems, and can be modeled with high accuracy. More broadly, they reinforce that post-collection storage is not a neutral holding phase, but a dynamic window of ecological change with profound implications for microbiome data integrity and implications for reproducibility, comparability and multi-omic data interpretation.

Importantly, shifts in metabolome profiles preceded microbial community restructuring, and temperature—not time—was the primary lever. In the sourdough model at 4 °C, the core sourdough bacterium *Fructilactobacillus sanfranciscensis* remained dominant throughout, reflecting its cold tolerance and efficient sugar utilization in mildly acidic environments^37^, and metabolite profiles remained relatively stable over time. In contrast, higher temperatures accelerated substrate depletion—particularly maltose and sucrose—triggering a succession from LAB to more acid- and storage stress-tolerant taxa such as *Acetobacter cerevisiae|malorum*, consistent with their known higher acid tolerance and metabolic flexibility^40–43^. This trajectory exemplifies an environmental filtering process^44^, wherein acid accumulation and resource scarcity seem to shift the community from sourdough specialization toward resilience and higher generalism. In line with this, *F. sanfranciscensis*, with its smaller genome and narrower metabolic repertoire^45^, declined with increasing storage temperature and time (Fig. 2e). A methodological consideration is that our species labels are derived from short-read marker-gene data. Short-read 16S rRNA V4 amplicon sequencing provides limited species-level resolution, and taxonomic assignments are often most reliable at the genus or family level, depending on the clade^46^. However, in our system, bacterial richness was low (maximum of eight OTUs/ASVs; Fig. 2a, Fig. S5a), and the classifier’s species-level labels matched canonical sourdough LAB species that have been described as prevalent and abundant across diverse sourdough ecosystems^14^. We further evaluated the robustness of our assignments by combining a SILVA 138.2 average-weighted Naïve Bayes classifier with manual blastn validation of the *Pediococcus* and *Acetobacter* ASVs which had lower classification confidence (below c = 0.7) against type-strain 16S rRNA sequences (Supplementary Fig. S16, Supplementary Tables S3–S4). Nevertheless, given the inherent limits of the 16S rRNA gene for bacterial classification and the dynamic nature of bacterial taxonomy, we treat the species labels as indicative but well supported within the sourdough niche and emphasize that our analyses and conclusions are robust to re-aggregation at the genus level, since the observed bacterial taxa belong to distinct genera.

These observed compositional transitions were likely driven by a combination of ecological stressors and metabolic interdependencies. As primary sugars (maltose, sucrose) were rapidly depleted—particularly under warmer storage—acidogenesis intensified, and acetic acid accumulated (Fig. 2d, Fig. S1a). Acetic acid, due to its membrane permeability and higher dissociation constant, imposes greater intracellular stress than lactic acid, which can promote decline of acid-sensitive LAB such as *F. sanfranciscensis* and facilitating the expansion of more acid-tolerant taxa like *A. cerevisiae|malorum*^27,47^. In parallel, metabolites produced by LAB and yeasts—such as lactic acid, ethanol, and amino acids—likely fostered cross-feeding by AABs, in line with established models of microbial succession involving AABs in other fermentation ecosystems^48^.

In parallel, untargeted FIA-MS metabolomics revealed a temperature- and time-dependent enrichment of nucleotides and stress-associated lipid classes (e.g., glycerophospholipids), indicative of nucleic acid degradation, membrane remodeling, and microbial turnover (Fig. 5b–d). The accumulation of select amino acids likely reflects both proteolytic activity and metabolite recycling under nutrient-depleted, acidified conditions^49^. Notably, glucose and fructose re-emerged at later timepoints (Fig. S3c), possibly released via endogenous carbohydrate breakdown or also via microbial lysis. These catabolic byproducts may have enabled the metabolic expansion of *A. cerevisiae|malorum*, which not only exhibits high stress tolerance but, as observed in kefir systems^48^ may also represent a late-successional taxon in the sourdough ecosystem that capitalizes on nutrient landscapes shaped by earlier fermenters.

Despite pronounced metabolic and bacterial shifts, fungal community structure remained remarkably stable across conditions (Figs. 4, 5), highlighting a functional decoupling between taxonomic stability and functional activity. This supports the notion that community composition does not always predict ecosystem function—a phenomenon previously reported in, e.g., marine microbiomes^50^. Importantly, our warmest and longest storage condition (30 °C for 28 days) was designed as an extreme upper-bound “stress test” for microbiome and metabolome stability rather than as a realistic or safe storage scenario for dough intended for consumption. The unchanged fungal evenness and generally the relatively stable fungal community structure at this condition should therefore not be interpreted as evidence of microbiological safety.

Under the studied storage conditions, the compositional decoupling was asymmetric: in bacterial communities, structural and functional profiles became more tightly coupled under warm, nutrient-limited conditions. Procrustes analyses revealed the strongest alignment between bacterial composition and metabolome profiles at 17 °C and 30 °C (M² ≈ 0.44–0.59; Fig. 5a), suggesting that under increased stress, microbial structure becomes a better predictor of function. This tighter coupling may arise from the erosion of functional redundancy under stress conditions.

Simultaneously, starvation stress exposure increased bacterial beta-dispersion (PERMDISP p < 0.001; Fig. 4), consistent with greater stochasticity and adaptive bottlenecks^51,52^. These shifts imply that while function becomes more predictable from community structure under stress, the structure itself becomes more variable between replicates. Temperature effects explained progressively more variance in Bray-Curtis dissimilarity (nested PERMANOVA R²: 30 °C > 17 °C > 4 °C), underscoring the dominant role of environmental filtering in shaping microbial ecology during storage.

Multi-omics network analyses revealed profound rewiring of ecological interaction topologies in response to storage temperature. At 4 °C, networks exhibited a modular architecture with *F. sanfranciscensis* maintaining high abundance but lower connectivity, consistent with niche partitioning and community stability under cold, sugar-rich conditions (Fig. 6c, f; Fig. S9, S10). In contrast, elevated temperatures led to a collapse of modularity and emergence of centralized structures, where *A. cerevisiae|malorum* assumed a hub-like role with high betweenness and eigenvector centrality. This transition likely illustrates a stress-induced reorganization from distributed, niche-driven coexistence to centralized ecological dependence, with *A. cerevisiae|malorum* functioning as an interaction keystone—likely mediating metabolite fluxes or coordinating inter-kingdom dynamics, supporting its previously suggested metabolic importance when studied in synthetic sourdough co-cultures^53^.

These shifts were uncovered through integrated, multi-layer network models that capture both positive and negative associations among bacteria, fungi, and metabolites. Unlike single-layer approaches, these frameworks reveal both abundance correlations and structural embedding—linking taxa to metabolite turnover and ecological roles. Notably, *A. cerevisiae|malorum*’s increased centrality did not merely reflect growth, but highlighted its functional anchoring as a metabolic and ecological mediator within the network at elevated temperatures. This underscores how community dominance under, e.g., stress conditions, can emerge not only from abundance, but from a taxon’s network position and embeddedness. Such multi-domain integration aligns with recent advances in gut microbiome research^54^, and offers a powerful framework for investigating resilience and stability also in fermented food ecosystems.

Building on this systems-level view, we next explored how predictive signatures of storage conditions are embedded within multi-omics profiles. Our supervised learning approach revealed that metabolite features were disproportionately sensitive to storage conditions: 24 of the top 25 predictors of storage temperature and duration were derived from the FIA-MS metabolome (Fig. 7b). This underscores the heightened vulnerability of functional layers to post-collection drift. Metabolomic data thus not only reflect system function but also encode metadata signatures of handling history and post-collection storage bias. Such sensitivity, while a source of distortion, also provides an opportunity—these molecular signatures could serve as proxies for sample integrity or environmental exposure.

These findings carry critical implications for microbiome studies relying on decentralized sampling and transport. Even brief exposure (1–2 days) to elevated temperatures triggered measurable changes in metabolite composition, bacterial evenness, and viability, while prolonged exposure (from 5 to 6 days onwards) induced divergence in community structure and ecological networks (Figs. 2–6). Notably, microbial succession lagged behind metabolome remodeling, underscoring the heightened sensitivity of the metabolome to handling conditions—and its potential utility as an early marker of post-collection perturbation.

Such storage-induced biases can distort ordination and clustering, mislead feature selection, and undermine the accuracy of downstream machine learning models—especially when unrecognized. Our findings echo concerns raised in other microbiome contexts, including human gut studies, where technical artifacts have been shown to dominate biological signals if uncorrected^16,55,56^. These observations emphasize the urgent need for rigorous metadata tracking, awareness of post-collection shifts, and the development of standardized correction workflows—particularly in large-scale, decentralized food microbiome efforts. A limitation of our experimental design is that storage temperatures were held constant, whereas real-world shipping and home-storage conditions often involve repeated temperature fluctuations and short-term temperature increases or decreases^57^. Our framework nevertheless captures the dominant directional effects of cooler versus warmer storage on community and metabolite trajectories and can be readily extended in future work to controlled fluctuating time–temperature profiles, as for example derived from empirical cold-chain measurements.

In conclusion, our study combines high-resolution storage tracking with machine learning–driven modeling in a controlled sourdough ecosystem to illuminate how microbial and metabolic profiles shift during post-collection storage. Through integrative, multi-omics analysis, we present a scalable, generalizable framework for quantifying and modeling collection-induced bias in microbiome research, and sourdough proved again its value as a tractable model for studying ecological and functional dynamics under environmental stress. Critically, the strong predictive signals encoded in storage-altered multi-omics profiles offer a new avenue for detecting—and potentially correcting—post-collection drift. This opens the door to more accurate, bias-aware interpretation and comparability of microbiome multi-omics data from decentralized, large-scale studies across diverse systems.

## Methods

### Fermentation experiments

To simulate transportation time and seasonal temperature variation, sourdough samples were stored at three constant temperatures 4 °C, 17 °C, and 30 °C that bracket typical refrigerated and ambient storage conditions, and sampled at 11 time points over one month (daily for the first 7 days, then on days 10, 14, 21, and 28). For sample sourdough preparation, we first established a laboratory wheat sourdough starter. Briefly, 10 g organic wheat flour (L170524, Swissmill, CH-8037 Zurich) were mixed with 10 g autoclaved, deionized and filtered water in a sterile 100 mL glass beaker. Autoclaved, deionized and filtered water was used instead of tap water to minimize variability in initial microbial inputs from the water supply and to standardize water composition. The mixture was covered with a loose aluminum foil cap to allow air circulation and incubated at 20 °C and 80% relative humidity for 24 h in a climate chamber (Memmert Peltier incubator). The starter was then refreshed for 14 consecutive days by removing 10 g of fermented dough and adding 5 g fresh flour and 5 g autoclaved, deionized water. After these initial 14 days, the starter was back-slopped once per week, with each 24 h fermentation step followed by storage at 4 °C until the next refreshment.

This laboratory-prepared wheat sourdough starter was subsequently upscaled for use in the storage experiment. In a first upscaling step, the starter (dough yield 200) was expanded using 500 g of organic wheat flour and 0.5 L autoclaved, deionized water under the same fermentation conditions as above. After 24 h, this sourdough was further upscaled overnight to a total of 8 kg. The 8 kg batch was distributed across five containers (each inoculated with 200 g of the same mother sourdough, 700 g water, and 700 g flour; incubated at 20 °C and 80% relative humidity), homogenized, and then divided into 99 aliquots (Gosselin Straight Containers, Fisher Scientific AG, Cat. No. 15458824) of 75 g each to cover the three storage temperatures and 11 sampling points. Samples were incubated with closed lids until harvest. A sample of the mother sourdough was collected at day 0, immediately after the overnight upscaling at 20 °C and before aliquoting into individual containers. The measurements of this day 0 sample are included in the supplements (Fig. S1, Fig. S4, S5 and S8) for comparison, but were not included in the main statistical analyses, because the sample represents one batch (the ‘mother’ dough, no biological replicates), was fermented at 20 °C rather than at the three experimental storage temperatures, and has a different effective fermentation history than the subsequent 24 h storage intervals.

### Sample collection and processing

At each time point, three aliquots per temperature condition were randomly selected for microbiological and chemical analyses. All samples (*n* = 99), including the baseline sourdough (day 0), were diluted 1:1 in molecular-grade water and stored at −80 °C for subsequent DNA and metabolite extraction. For microbiological analysis, 5 g of sourdough was homogenized with 45 g of 1% vegetable peptone (VWR International, Cat. Nr. OXOIVG0100B) and 0.85% NaCl solution, then serially diluted. Viable lactic acid bacteria (LAB) counts were determined by plating on De Man, Rogosa, and Sharpe 5 (MRS-5) agar^58^, prepared following their exact recipe containing 0.1 g/L cycloheximide, except exchanging meat extract with Beef Extract 500 (Merck, Cat. Nr. B4888-500G), and peptone with vegetable peptone (VWR International, Cat. Nr. OXOIVG0100B). Viable yeast counts were determined by plating on Yeast Extract Peptone Glucose (YPG) agar supplemented with 0.2 g/L chloramphenicol. Plates were incubated aerobically at 30 °C and 80% relative humidity for 48 h before calculating the number of colony-forming units (CFU) per gram. For chemical analysis, 10 g of sourdough was homogenized with 100 g of non-sterile deionized water. The pH was measured immediately, and total titratable acidity (TTA) was quantified by titrating with 0.1 M NaOH to a pH endpoint of 8.5, following the exact procedure as reported in the HealthFerm sourdough citizen science initiative^15^.

### Substrate removal from sourdough samples and DNA extraction

DNA was extracted using the MagMAX™ Microbiome Ultra Nucleic Acid Isolation Kit (Thermo Fisher Scientific) on the KingFisher Apex platform (Thermo Fisher Scientific), following the MagMAX Liquid Buccal protocol (Thermo Fisher Scientific) with minor modifications. Cryotubes containing sourdough aliquots were thawed on ice, randomized, and transferred to KingFisher 96 deep-well plates. ZymoBIOMICS Microbial Community Standard (37.5 μL diluted in 112.5 μL sterile water; Zymo Research, Cat. No. D6300) and sterile H₂O (150 μL) served as positive and negative extraction controls, respectively. To prevent yeast sedimentation, samples were briefly homogenized at 20 Hz using a TissueLyser III (Qiagen). To remove flour particles while retaining larger yeast cells, plates were centrifuged at 100 × *g* for 1 min. A 150 μL aliquot of the resulting supernatant was transferred to the MagMAX Bead Beating Plate. Bead beating was performed twice for 5 min at 30 Hz, and DNA was eluted in 50 μL of elution buffer. DNA concentrations were measured using the Qubit™ dsDNA Quantification Assay Kit (Thermo Fisher Scientific, Cat. No. Q32854), following the manufacturer’s instructions. DNA was stored at −20 °C until further processing.

### Marker gene amplicon library preparation and sequencing

Amplicon libraries for bacterial (16S rRNA gene) and fungal (ITS) profiling were prepared using the HighALPS ultra-high-throughput protocol with unique dual indices (UDI)^59^. Bacterial communities were amplified targeting the V4 region using UDI-linked primers 515 F (5′-GTGYCAGCMGCCGCGGTAA-3′) and 806 R (5′-GGACTACNVGGGTWTCTAAT-3′)^60,61^. Reactions (25 μL) contained 5 μL of template DNA, 12.5 μL 2× KAPA HiFi HotStart ReadyMix (Roche, Cat. No. 07958935001), and 0.3 μM primers. PCR conditions were: 95 °C for 5 min; 35 cycles of 98 °C for 20 s, 55 °C for 15 s, and 72 °C for 25 s; followed by a final extension at 72 °C for 4 min.

Fungal communities were profiled via nested PCR targeting the ITS1 region using BITS (5′-ACCTGCGGARGGATCA-3′) and B58S3 (5′-GAGATCCRTTGYTRAAAGTT-3′) primers^62^. The first round used non-indexed primers (25 μL reactions with 2 μL DNA template), followed by barcoding PCR using UDI-tagged primers with 1 μL of the first reaction as input. All primers were ordered from Microsynth AG, Switzerland. Cycling conditions were: 95 °C for 5 min; 35 cycles of 98 °C for 20 s, 49 °C for 15 s, and 72 °C for 20 s; with a final extension at 72 °C for 4 min. Barcoding PCR consisted of 10 cycles under the same thermal profile.

Amplicons were purified using Agencourt AMPure XP magnetic beads (0.7× ratio; Beckman Coulter, Cat. No. A63882) on the KingFisher Apex platform. DNA concentrations were quantified in duplicate using the Qubit™ dsDNA High Sensitivity Assay (Thermo Fisher Scientific, Cat. No. Q32854) on a Tecan Spark microplate reader. 16S rRNA and ITS amplicons were pooled separately at equimolar concentrations using a liquid handling platform (Brand GmbH), and quality was assessed via high-sensitivity TapeStation (Agilent Technologies, High Sensitivity D1000 DNA ScreenTape assays, Cat. No. 5067-5584). Pooled libraries were combined and sequenced with 300 bp paired-end reads on the Illumina NextSeq 2000 (600-cycle kit), including a 20% PhiX spike-in, at the Functional Genomics Center Zürich.

### Quantitative PCR

Quantitative PCR (qPCR) was used to determine total, viable and non-viable, bacterial (LAB and non-LAB) abundance across all storage conditions. Each 25 μL reaction contained 1 μL of template DNA, 12.5 μL of 2× KAPA HiFi HotStart ReadyMix (Roche, Cat. No. 07958935001), 1 μL of 20× EvaGreen Dye (Brunschwig, Cat. No. BIO31000), and 0.3 μM of non-barcoded 515 F/806 R primers. Thermal cycling was performed on a LightCycler 480 (Roche) with the following conditions: 95 °C for 3 min; 40 cycles of 98 °C for 20 s, 55 °C for 15 s, and 72 °C for 15 s; followed by melt curve analysis (95 °C for 30 s, 60 °C for 15 s, ramping at 1 °C/min). Absolute DNA concentrations (ng/μL) were determined using a standard curve generated with the ZymoBIOMICS Microbial Community DNA Standard (Zymo Research, Cat. No. D6305), and applied to cycle threshold (Ct) values from sourdough samples. Bacterial cell counts were estimated assuming an average of five 16S rRNA gene copies per cell and 1.58 × 10⁹ 16S rRNA gene copies per ng of DNA (Table S1).

### HPLC sample preparation and measurement

Pre-diluted sourdough samples stored at −80 °C were thawed on ice for 30–45 min. A 200 mg aliquot was diluted 1:10 in LC-MS grade water (Merck Supelco), homogenized in a Thermomix at 1500 rpm and 10 °C for 10 min, and centrifuged at 14,000 × *g* and 4 °C for 15 min. The resulting supernatant was filtered through a 0.45 μm polyvinylidene difluoride (PVDF) membrane (BGB Analytik AG) to remove particulates. For organic acid and ethanol analysis, 200 μL of filtered supernatant was transferred into glass HPLC vials with inserts. For sugar analysis, 100 μL of supernatant was further diluted 1:10 with Milli-Q water (MilliporeSigma) in plastic vials. Samples were stored at 4 °C for up to 48 h or at −20 °C for longer-term storage.

#### Organic acid and ethanol detection

Organic acids (succinic acid, lactic acid, acetic acid) and ethanol were quantified using an Agilent 1200 Series HPLC system equipped with an Aminex HPX-87H column (Bio-Rad). The column was maintained at 40 °C with a sample tray temperature of 10 °C and an injection volume of 10 μL. Separation was achieved under isocratic conditions using 5 mM sulfuric acid (H₂SO₄; Merck Titrisol) at a flow rate of 0.4 mL/min over 40 min. Ethanol was detected via refractive index (RI) detection at 40 °C, while organic acids were quantified using a diode array detector at 210 nm.

#### Carbohydrate detection

Carbohydrates (mannitol, sucrose, glucose, fructose, maltose) were analyzed using a Dionex ICS-5000+ system (Thermo Scientific) equipped with a Dionex CarboPac PA200 IC column maintained at 25 °C. Samples were held at 10 °C and injected at 10 μL. Separation was performed at a flow rate of 0.25 mL/min using a three-solvent system: (1) 225 mM sodium hydroxide (NaOH), (2) 1250 mM sodium acetate with 100 mM NaOH, and (3) Milli-Q water. A defined gradient program (Table S2) was applied, and carbohydrates were detected via pulsed amperometric detection. Total runtime per sample was 45 min.

### FIA-MS untargeted metabolomics

All steps were performed on ice using ice-cold solvents unless otherwise specified. For metabolite fingerprinting via flow injection analysis-mass spectrometry (FIA-MS), 100 μL of water extract (from the HPLC organic acid analysis; see above) was diluted with 900 μL LC-MS grade methanol (Fisher Chemical), vortexed briefly, and centrifuged at 4500 × g for 30 min at 5 °C. The supernatant was further diluted 1:10 in 50% methanol, mixed, and 150 μL was transferred to a 96-well plate, heat-sealed with foil (Thermo Fisher Scientific), and analyzed within 24 h. FIA-MS was conducted using an Acquity I-Class UPLC system coupled to a Xevo G2-XS qToF mass spectrometer (Waters), following a modified protocol from Fuhrer et al.^63^. Two microliters of extract were injected in 60% methanol containing 0.05% ammonium hydroxide (NH₄OH, Honeywell) and 2 mM ammonium fluoride (NH₄F; Sigma-Aldrich) as carrier solvent, at 0.2 mL/min for 1 min. Compounds were ionized using electrospray ionization in negative sensitivity mode with the following source parameters: capillary voltage 2.2 kV, cone voltage 40 V, source offset 80 V, source temperature 120 °C, desolvation gas at 250 °C and 850 L/h, and cone gas at 150 L/h. Mass spectra were acquired in extended dynamic range from m/z 50–1200 at a scan rate of 0.7 s. For online mass correction, 200 ng/μL Leucine-Enkephalin ([M–H]⁻ 556.2771; Waters) in 50:50:0.1 acetonitrile:water:formic acid was injected every 19 s (scan time 0.3 s, capillary voltage 2.2 kV).

Raw data were converted to mzML format using MSConvert v3.0 (ProteoWizard)^64^, and processed in MZMine 4.1.0^65^. Mass detection used centroid mode with a noise level of 1000. Chromatogram building used the ADAP algorithm (min scans: 4, min group intensity: 2000, min absolute height: 2000, m/z tolerance: 0.005 or 10 ppm). Chromatograms were smoothed with a Savitzky–Golay filter (RT window: 5). Carbon-13 deisotoping applied intra-sample tolerances of 0.002 m/z or 3.5 ppm and 1 min RT. Feature alignment used the Join Aligner (m/z tolerance: 0.005 or 15 ppm; m/z weight: 80; RT tolerance: 1 min; RT and mobility weights: (1). Gap filling used an intensity tolerance of 0.2, m/z tolerance of 0.005 or 15 ppm, and a minimum of 4 scans. Blank subtraction removed features with intensity <5x that of corresponding blanks. Final exported peak heights were used for statistical analysis.

Ions were annotated by exact mass (m/z) matching (±0.002 Da) against the KEGG metabolite^66^ and HMDB databases^67^, and subsequently classified using the HMDB metabolite taxonomy. Annotated features were then grouped into compound classes or subclasses relevant to sourdough fermentation (e.g., amino acids, organic acids, carbohydrates) for visualization. These groupings were used to calculate and plot the relative proportions of annotated metabolites within each temporal response cluster and temperature condition (Fig. 4).

### Bioinformatic marker gene amplicon processing

#### Fungal ITS amplicons

Raw paired-end internal transcribed spacer (ITS) sequences were processed using QIIME 2 version 2024.10^68^. Primers and adapters were trimmed with the cutadapt trim-paired plugin^69^. Denoising was performed via dada2 denoise-single plugin^70^ with no truncation (--p-trunc-len 0), a maximum expected error of 4.0, and a minimum parent abundance fold-difference of 4.0, resulting in 96–98% non-chimeric reads. Reads shorter than 50 nt were filtered out, retaining 2,453,636 sequences across 33 samples per temperature group (4 °C, 17 °C, 30 °C), one day 0 mother control, and excluding one contaminated replicate at day 7. Taxonomy was assigned with the classify-sklearn action of the q2-feature-classifier plugin^71^ against the customized UNITE v10.99 reference database^72^ curated via RESCRIPt^73^; initial classification followed a confidence threshold of 0 to retain low-confidence fungal reads (to enable filtering of off-target hits as described below), followed by classification with the default confidence threshold of 0.7 for high-confidence classification of on-target hits^71^. Species labels reported in the manuscript were assigned based on the default 0.7 confidence threshold. Reads assigned to non-fungal sequences, low-abundance features (<10 reads/sample), fruiting body–associated taxa, unclassified phyla, and extremely rare class-level taxa were removed, accounting for 1.4% of total reads and resulting in 2,419,268 reads. Operational taxonomic units (OTUs) were generated by mapping reads at 90% similarity using the vsearch cluster-features-closed-reference plugin^74^, against the same UNITE v10.99 database, excluding singletons. Unmatched and poorly classified reads were discarded, yielding a final OTU table of 2,411,351 reads—a 0.33% reduction from the ASV set. Both ASV and OTU tables were rarefied to 3500 reads per sample, resulting in a final dataset of 95 samples (Fig. S14a, b).

#### Bacterial 16S rRNAv4 amplicons

Paired-end 16S rRNAv4 gene sequences were processed using the dada2 denoise-paired plugin in QIIME 2. Reads were truncated at 150 bp in both directions to remove low-quality regions, primers, and adapters. Denoising used dada2 (via the q2-dada2 plugin) with a maximum expected error threshold of 2.0 and a minimum fold-parent-over-abundance threshold of 4.0, yielding 84–86% non-chimeric reads and a total of 4,019,885 denoised, merged sequences with a mean length of 252.98 ± 3.48 bps (consistent with an expected length of ~250 bps for the EMP protocol). One sample (day 28 at 4 °C) failed, resulting in 32 samples for 4 °C, 33 for 17 °C, 33 for 30 °C, and 1 mother sourdough control. All retained reads were ≥50 bp, so no additional length filtering was required. Taxonomic classification was performed using the classify-sklearn plugin trained on a custom SILVA 138.2 SSU NR99 reference database 75, restricted to EMP 515 f/806r amplicon regions and prepared using RESCRIPt, using a staged classification approach as described above. Initial indicative classifications for quality control used a confidence threshold of 0 to maximize bacterial read retention for quality filtering purposes, filtering out all non-bacterial sequences (e.g., mitochondria, chloroplasts, archaea, eukaryotes), which accounted for 13.46% of reads. An additional 492 ASVs were removed via contaminant filtering^75^ (decontam-combined action of the q2-quality-control plugin) and removal of rare features (<10 reads in any sample), resulting in 3,478,383 high-quality reads. Reads were additionally clustered into OTUs at 99% identity using the vsearch cluster-features-closed-reference plugin against SILVA 138.2, yielding a final OTU table with 3,478,356 reads (only 27 fewer than the ASV table due to unmatched sequences). Both ASV and OTU tables were subsequently rarefied to 380 reads/sample, resulting in a final dataset of 93 samples (Fig. S14c, d). Final taxonomic classification of the filtered ASVs/OTUs was performed using a pre-trained SILVA 138.2 SSU NR99 reference database^76^ “average weighted” naive Bayes classifier, which uses ecological frequency information as class weights to improve taxonomic classification to most probable species^77^ (Fig. S16a–c). Among the top 8 most abundant ASVs, which collectively encompass 100% of the total relative frequency across all samples, taxonomic classifications were manually checked to confirm species labels via local alignment with blastn^78^ against the NCBI RefSeq 16S database^79^ (Fig. S16d, e; Tables S3, S4); this approach was used to confirm identification of *Pediococcus parvulus* and *Acetobacter cerevisiae|malorum* (the latter share 99.9% full-length 16S rRNA gene similarity and hence the exact species cannot be resolved with the 16S v4 fragment). Nevertheless, given the limited resolution of the 16S rRNA gene for bacterial classification, and the dynamic nature of bacterial taxonomy, all species-level classifications included here should be interpreted as indicative rather than definitive.

### Statistical analysis

#### Microbial diversity analyses

Diversity analyses, including calculations of alpha-diversity (richness, evenness and Shannon entropy) and beta-diversity (Jaccard distance and Bray–Curtis dissimilarity), were made using the q2-diversity and q2-kmerizer^80^ plugins in QIIME 2. Beta diversity estimates were calculated based on ASV composition as well as the constituent k-mers in these ASVs, and OTU features.

#### FIA-MS features preprocessing and clustering

FIA-MS features were filtered with a prevalence threshold of 3 (triplicates) and missing values were imputed as zeros. Feature tables were processed using z-score normalization, log transformation, combined log-z transformation, or total sum scaling (TSS). To capture dynamic metabolite responses to storage, one-way ANOVA (α = 0.05) was performed across time points within each temperature group on z-score normalized data. Metabolites showing significant temporal variation in at least one temperature condition were retained, and non-responsive features were excluded prior to clustering. Filtered, z-score normalized metabolite features were clustered using k-means in scikit-learn^81,82^. Optimal cluster number was determined via silhouette score, elbow method (WCSS), and Gap statistic. Final clusters were defined with the best-supported k (k = 3) across all evaluation metrics. For each cluster, temporal dynamics were visualized by plotting z-score trajectories per temperature. Kruskal–Wallis tests were performed per cluster and day to assess temperature-driven differences, with Benjamini-Hochberg correction applied to control for false discovery rate.

#### Two-dimensional feature space statistics

Microbial beta-diversity feature spaces were visualized by using principal coordinate analysis (PCoA) and FIA-features by using Principal Component Analysis (PCA) to reduce the dimensionality. To aid interpretation of sample separation in beta-diversity PCoAs and FIA PCAs, top correlated features and metadata variables were overlaid in feature space. Pearson correlations between each variable and the first two PCoA respectively PCA axes were used to determine direction and length. Top contributors were visualized as scaled vectors, highlighting features and metadata most associated with ordination structure. To evaluate the contributions of experimental factors and their nested or interacting effects on community and metabolomic dissimilarity structures, we applied the QIIME 2 diversity adonis plugin for global PERMANOVA modeling (Fig. S7a, d, g) and a custom two-level nested PERMANOVA implementation in scikit-bio (Fig. S7b, c, e, f, h, i)^83–85^. For the adapted nesting, global variance was first assessed for primary factors (e.g., temperature or day), followed by separate within-group PERMANOVA tests on secondary factors (e.g., day nested within each temperature, or temperature nested within each day). p-values were corrected for multiple testing using the Benjamini–Hochberg false discovery rate (FDR) procedure.

#### Day-wise comparison with post hoc grouping

To assess temperature effects across time, one-way ANOVA was applied at each time point for continuous variables including alpha diversity (amplicon-based and FIA-MS), CFUs, pH, TTA, and HPLC-derived metabolite concentrations (excluding control samples). Post hoc comparisons were performed using Tukey’s Honest Significant Difference (HSD) test to identify temperature-specific differences per day. Prior to conducting ANOVAs, homogeneity of variance was evaluated using Levene’s test across temperatures for each time point and variable (Fig. S15). As no comparisons showed significant heterogeneity (p < 0.05), ANOVA assumptions were considered met despite small replicate numbers.

#### Differential abundance analysis

Microbial compositional shifts were tested using ANCOM-BC2^86^ in R v4.5.0. Amplicon data were rescaled to pseudo-counts and analyzed using a phyloseq-based workflow, excluding control (20 °C) samples. The model included day and temperature as fixed effects, and both pairwise and global tests were performed with FDR correction. Structural zeros and sampling fraction variation were accounted for. Taxa showing log-fold changes with a certain threshold (|logFC| > 0.75 for 16S, |logFC| > 0.1 for ITS) were classified as enriched (numerator) or outcompeted (denominator). Sample-wise log-ratios were computed as the log geometric mean of enriched taxa over depleted taxa at given temperatures and time points.

#### Integrations of metabolomics and amplicon data

To assess the concordance between metabolic and microbial community structures, Bray-Curtis dissimilarity matrices were computed on 16S OTU feature tables and subsequent PCoA, whereas FIA-MS (either z-score or TSS normalized) was either processed via Bray-Curtis and PCoA or direct PCA. Subsequently, Procrustes analysis was used to align ordination spaces between microbiome and metabolome data. To evaluate statistical significance, permutation tests (n = 1000) were conducted by randomizing sample labels in one dataset and recomputing Procrustes disparities, yielding empirical p-values. Disparities and associated p-values were computed for global comparisons, as well as stratified by day and temperature in isolation or stratification, to identify temporal or condition-specific shifts in metabolome–microbiome concordance.

#### Multi-omics co-occurrence analysis and network inference

To investigate intra- and inter-kingdom associations in the sourdough microbiome and metabolome across storage temperatures, we performed two complementary network inference approaches based on centered log-ratio (CLR)–transformed data. In the first approach, microbial OTU tables (16S and ITS) and metabolomics data were CLR-transformed (after multiplicative replacement; delta = 1e-8; with TSS normalization for metabolite data) and pairwise associations were calculated using a novel abundance-weighted co-occurrence score (Eq. 1).

1 $$Co-occurrence(x,y)=\frac{{\sum }_{i=1}^{n}{x}_{i}* {y}_{i}* {w}_{i}}{{\sum }_{i=1}^{n}{w}_{i}},where\,{w}_{i}=\left|{x}_{i}* {y}_{i}\right|$$

Equation (1) is inspired by compositional-aware correlation frameworks such as SparCC^87^ and CoNet^88^, and enhances traditional co-occurrence metrics by explicitly upweighting high-abundance, co-varying features while suppressing noise from low-abundance artifacts. Unlike SparCC, which infers correlations via iterative pseudo-count-based covariance estimation, or CoNet, which combines multiple similarity metrics with heuristic edge selection, our score directly integrates abundance information into the association weight, offering greater sensitivity to biologically meaningful, high-confidence co-occurrence in complex multi-omics datasets. Associations with ∣Score(x,y)∣ ≥ 0.2 were retained for downstream network construction. This approach supports robust detection of ecologically relevant interactions across kingdoms and omics layers, while maintaining interpretability and scalability for large datasets.

In parallel, we applied a sparse inverse covariance estimation approach to capture conditional dependencies and partial correlations among features. CLR-transformed feature matrices were z-score standardized and concatenated, followed by model fitting using the Graphical Lasso with cross-validation in scikit-learn^82,87^. Non-zero entries in the resulting precision matrix defined network edges, representing regularized partial correlations. Feature identity (bacteria, fungi, metabolite) was used to classify intra- and inter-domain interactions. The resulting co-occurrence matrices were visualized as undirected networks in NetworkX (v2.8.8)^89^, with node attributes encoding biological category (bacteria, fungi, metabolite), shape, and average abundance (used for node size scaling). Network layout was performed using Graphviz’s “neato” force-directed algorithm^90^. Edge thickness and spring length were scaled by co-occurrence strength, and edge color represented co-occurrence polarity. Topological metrics - degree centrality, betweenness centrality, closeness centrality, and eigenvector centrality - were calculated to identify key taxa and metabolites in each network. Networks and centrality metrics were generated independently for each storage temperature.

#### Supervised classification of storage conditions using multi-omics features

To predict sample storage temperature (4 °C, 17 °C, 30 °C), a supervised classifier was trained on combined microbial (16S OTU) and metabolomic (FIA-MS) feature matrices as well as HPLC, CFU, pH, TTA and DNA concentration (extractions) data. A nested pipeline was implemented using a random forest classifier^91^ (100 estimators) preceded by feature selection, which retained features with importances above the median threshold. Model evaluation was performed using stratified 5-fold cross-validation to preserve class balance per fold. This approach allowed robust performance estimation while accounting for both model fitting and feature selection within each training fold.

All statistical and computational analyses were performed in Python (v3.10.14), unless stated otherwise. Data preprocessing used pandas^92^ and numpy^93^. Dimensionality reduction and building and evaluating random forest models was performed with scikit-learn^82^. Random forest models were built and evaluated using scikit-learn. Statistical modeling (e.g., ANOVA) used statsmodels^94^. Visualizations were generated with Seaborn^95^, Matplotlib^96^ and NetworkX^89^.

## Supplementary information

Supplementary information

## Data Availability

All sequence data have been deposited on EBI-ENA under accession number PRJEB94514 (16S) and PRJEB94515 (ITS). Source data (metadata) along with processed HPLC and FIA-MS data have been deposited together with all code notebooks in github (see code availability).

## Code availability

All code notebooks for bioinformatic processing, statistical analyses, and machine-learning models have been deposited and are openly accessible in github https://github.com/bokulich-publications/shipped-and-shifted.
