# Supplementary material for: Shipped and shifted: modeling collection-induced bias in microbiome multi-omics using a tractable fermentation system
Source: NPJ Biofilms Microbiomes. 2026 Jan 9;12:43. doi: 10.1038/s41522-025-00909-1 (PMC12901149; doi:10.1038/s41522-025-00909-1)
Supplement: Supplementary file 1 — Supplementary information [file 41522_2025_909_MOESM1_ESM.pdf]

Supplementary information

Shipped and shifted: modeling collection-induced bias in microbiome multi-omics using a tractable fermentation system

Annina R. Meyer[1], Jan Patrick Tan[2], Mihnea Paul Mihaila[1], Michelle Neugebauer[1], Laura Nyström[2], Nicholas A. Bokulich[1#]

Affiliations

1. Laboratory of Food Systems Biotechnology, Department of Health Sciences and Technology, ETH Zürich, Schmelzbergstrasse 7, 8092 Zürich, Switzerland
2. Laboratory of Food Biochemistry, Department of Health Sciences and Technology, ETH Zürich, Schmelzbergstrasse 9, 8092 Zürich, Switzerland

#Correspondence

nicholas.bokulich@hest.ethz.ch

Supplementary figures

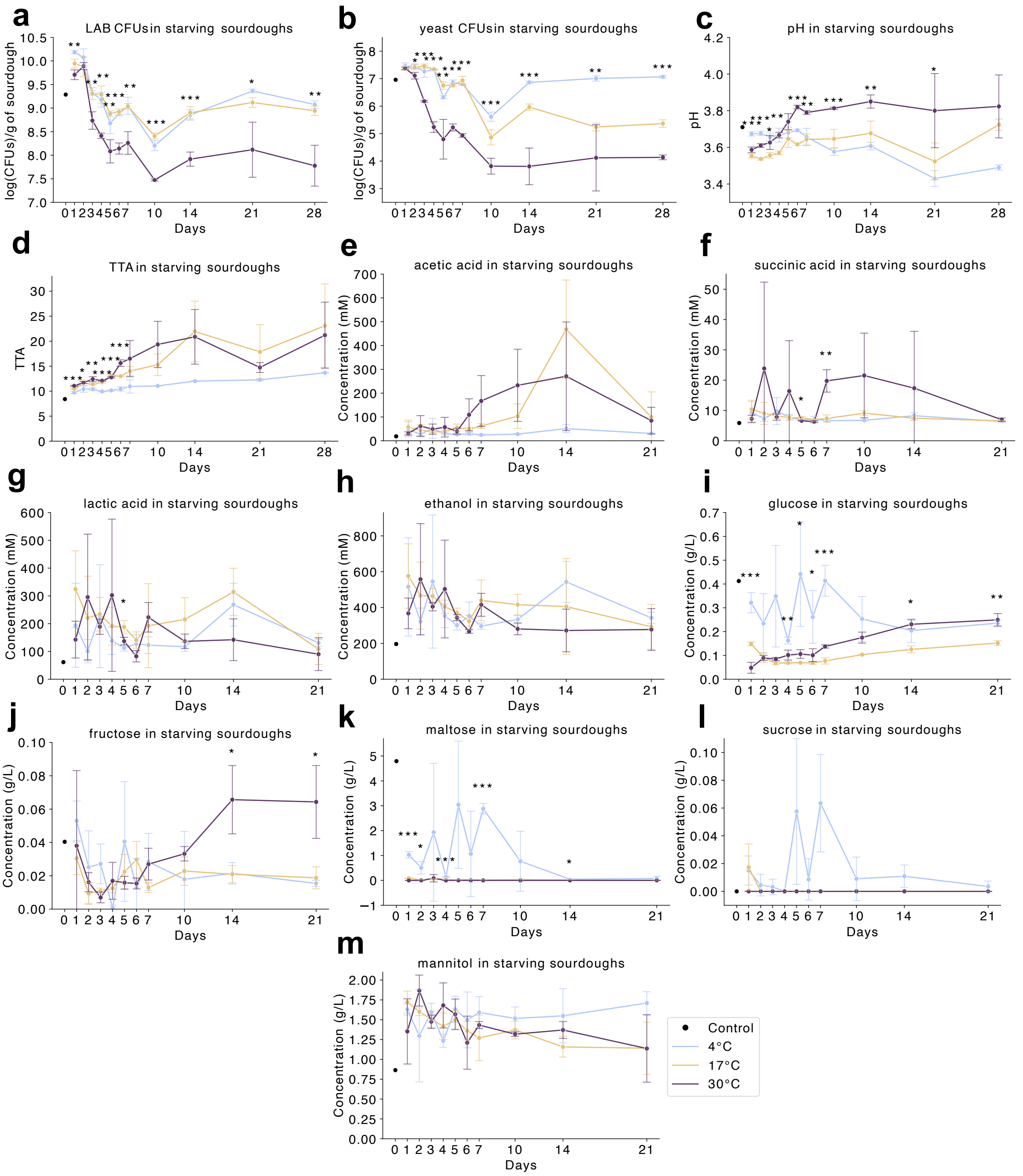

Supplementary Figure 1. Changes in microbial viability, total acidity, fermentation-relevant acids and sugars in sourdough samples stored at 4 °C, 17 °C, and 30 °C over 21 days. Viable lactic acid bacteria **(a)** and yeast **(b)** colony forming units (CFUs), pH **(c)** and total titratable acidity (TTA) **(d)** over time. HPLC-quantified metabolites (**e-m**) including acetic acid, succinic acid, lactic acid, ethanol, glucose, fructose, maltose, sucrose and mannitol. Statistical differences between storage temperatures at each time point were determined by one-way ANOVA with FDR correction (*$p < 0.05$, **$p < 0.01$, ***$p < 0.001$). Baseline measurements in the control ('mother') sourdough at day 0 (upscaled overnight at 20 °C before aliquoting) are shown as a filled black circle.

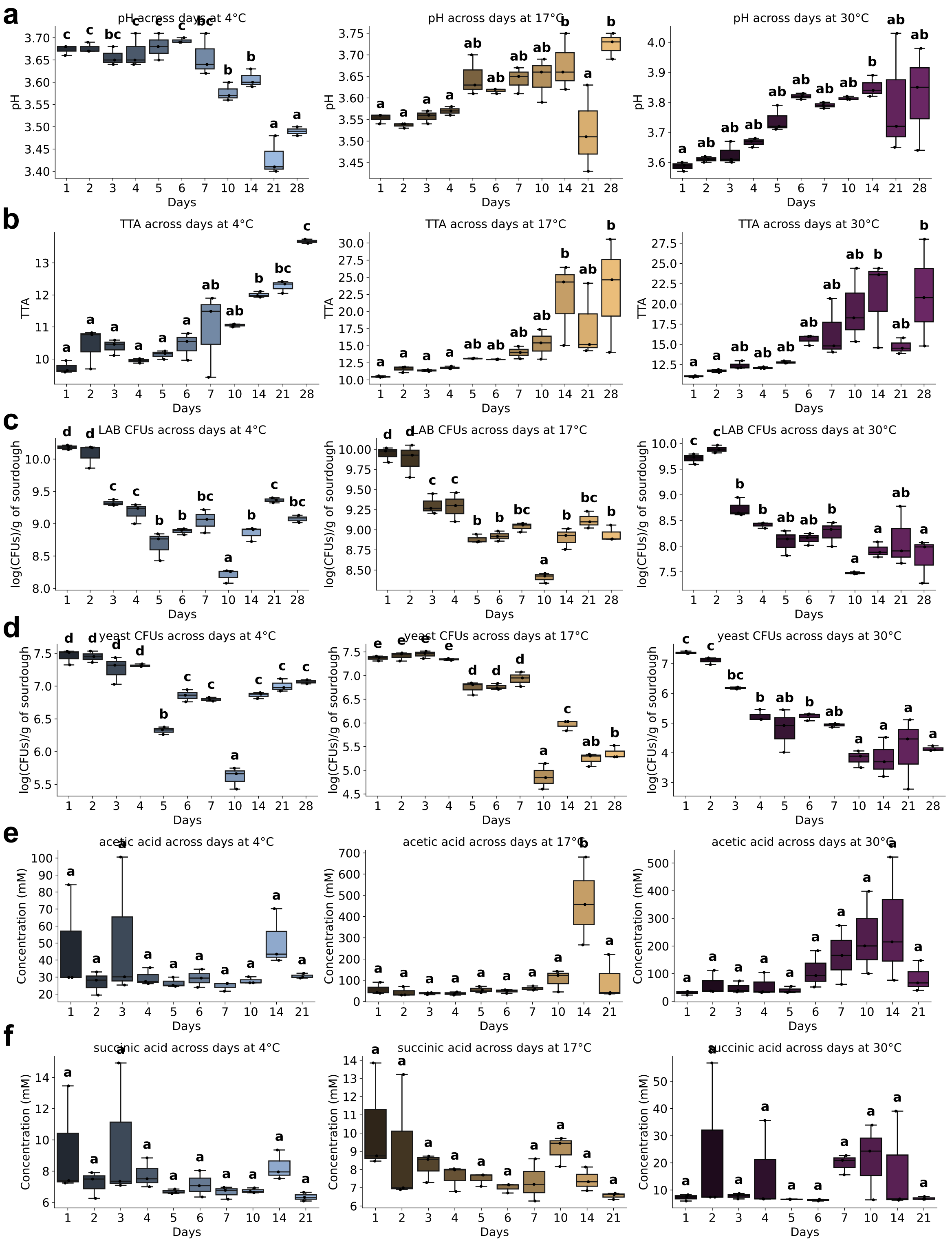

Supplementary Figure 2. Temporal dynamics of acidity, microbial viability, and organic acid accumulation in sourdough samples stored at 4 °C, 17 °C, and 30 °C. Measurements over the 28-day storage period are shown for **(a)** pH, **(b)** total titratable acidity (TTA), **(c)** viable lactic acid bacteria (LAB) counts, and **(d)** viable yeast counts; organic acid concentrations were monitored over 21 days for **(e)** acetic acid and **(f)** succinic acid. Statistical comparisons of time points within each temperature group were conducted using post hoc Tukey's HSD with FDR correction. Time points labeled with the same letter are not significantly different (adjusted $p > 0.05$), while those with different letters indicate significant differences (adjusted $p < 0.05$).

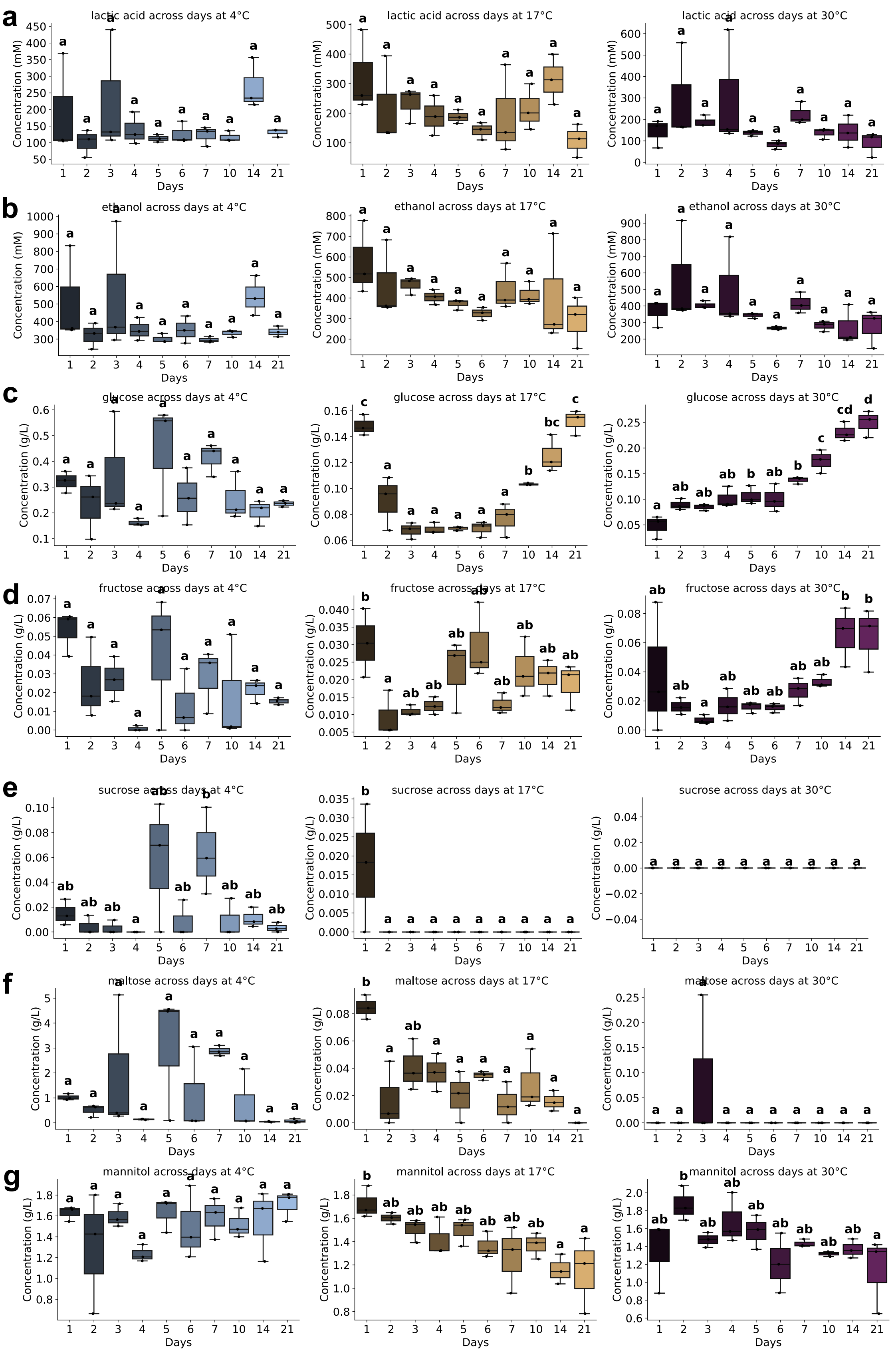

Supplementary Figure 3. Temporal dynamics of organic acid and sugar concentrations in sourdough samples stored at 4 °C, 17 °C, and 30 °C over a 21-day period. HPLC-quantified metabolites include: **(a)** lactic acid, **(b)** ethanol, **(c)** glucose, **(d)** fructose, **(e)** maltose, **(f)** sucrose, and **(g)** mannitol. Statistical comparisons of time points within each

temperature group were performed using post hoc Tukey's HSD with FDR correction. Time points sharing the same letter are not significantly different (adjusted $p > 0.05$), whereas those with different letters indicate statistically significant differences (adjusted $p < 0.05$).

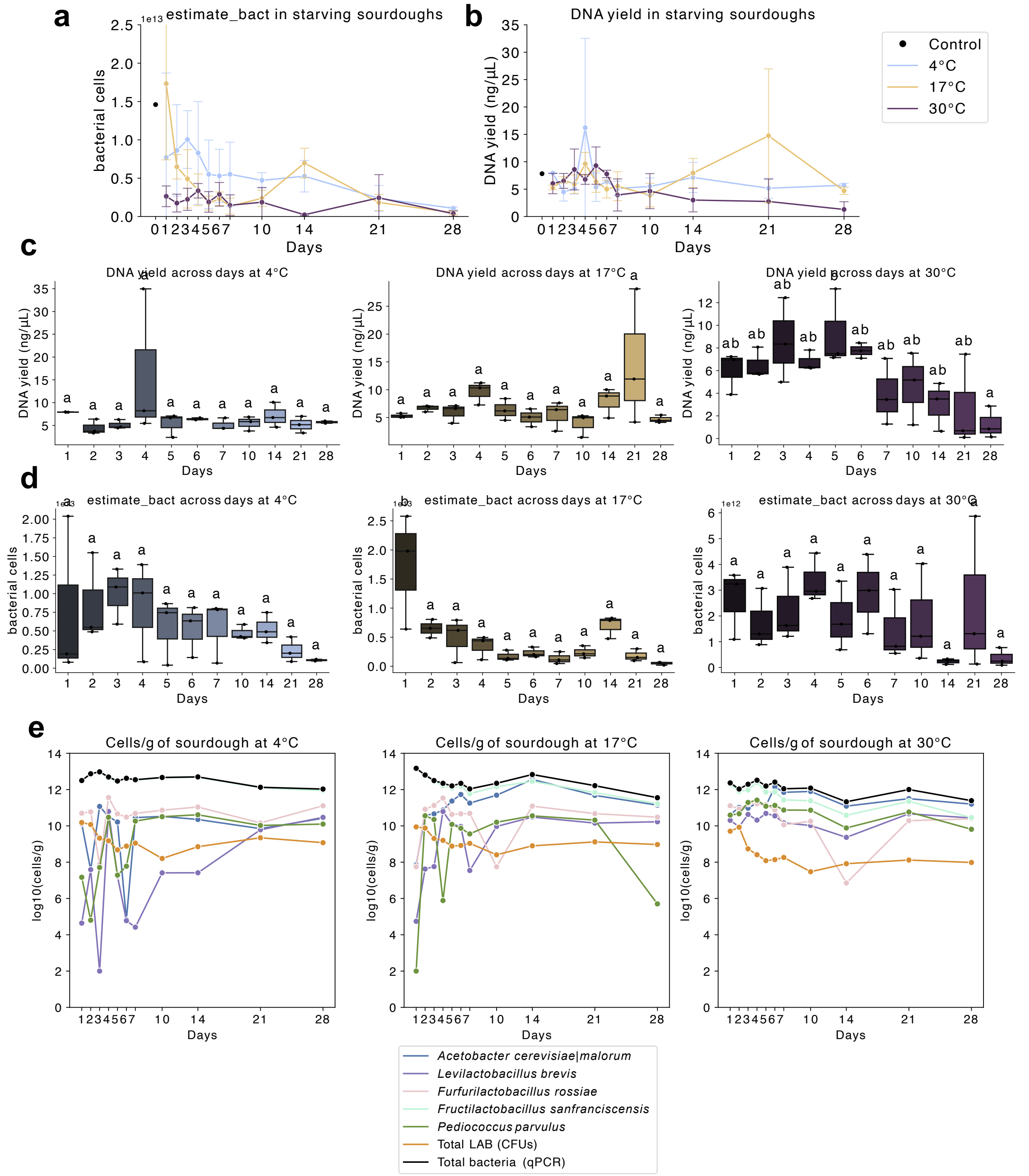

Supplementary Figure 4. Quantification of total bacterial load and DNA extraction efficiency in sourdough samples stored at 4 °C, 17 °C, and 30 °C over 28 days. a, Estimated total bacterial cells per gram of sourdough, determined by quantitative PCR (qPCR) targeting the V4 region of the 16S rRNA gene using EMP primers, normalized to the average 16S gene copy number in lactobacilli. **b,** DNA yield (ng/µL) obtained from extractions using the MagMAX Microbiome Ultra kit (Thermo Fisher). Statistical comparisons across temperatures at each time point (panels a, b) were performed using one-way ANOVA with FDR correction (*$p < 0.05$, **$p < 0.01$, ***$p < 0.001$). Baseline bacterial biomass estimation and DNA yield from the control ('mother') sourdough at day 0 (upscaled overnight at 20 °C before aliquoting) are shown as a filled black circle (**a,b**).Temporal comparisons within each

temperature group were conducted using post hoc Tukey's HSD with FDR correction for **(c)** DNA yield and **(d)** total bacterial cell estimates. Time points labeled with the same letter are not significantly different; those with different letters indicate significant differences (adjusted $p < 0.05$). **e,** Absolute abundances of dominant bacterial species over time, derived by multiplying 16S rRNA-based relative abundances with qPCR-derived total bacterial estimates. LAB colony-forming units (CFUs) are overlaid for comparative validation of viability versus total bacterial DNA abundance.

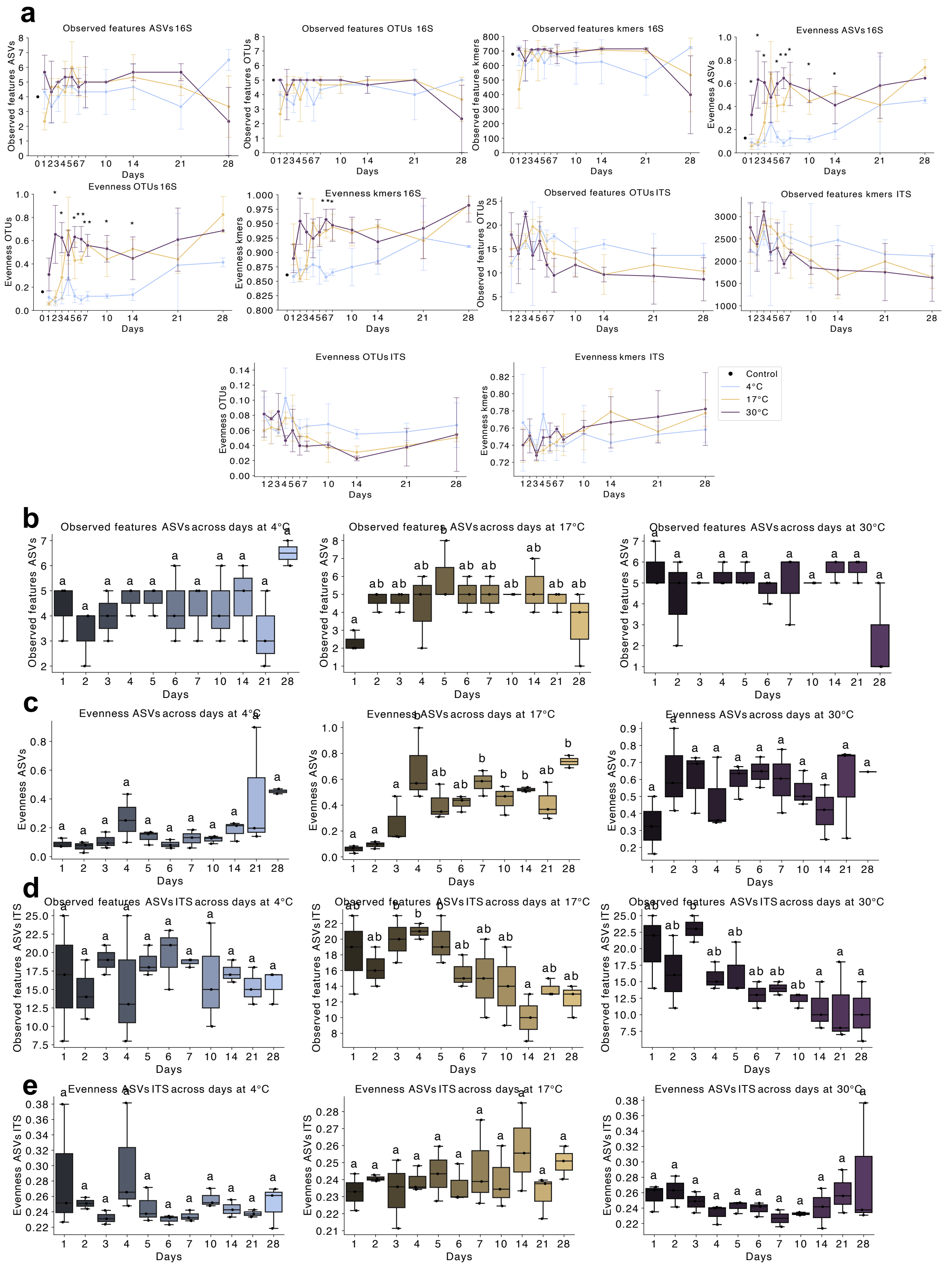

Supplementary Figure 5. Alpha diversity metrics of bacterial (16S V4) and fungal (ITS) communities in sourdough samples stored at 4 °C, 17 °C, and 30 °C over 28 days. a, Observed features and community evenness for bacteria and fungi, computed from both OTU clustering and k-mer–based analysis. Statistical comparisons across temperatures at each time point were conducted using one-way ANOVA with FDR correction (*p < 0.05, **p < 0.01, ***p < 0.001). Baseline bacterial alpha diversity from the control ('mother') sourdough at day 0 (upscaled overnight at 20 °C before aliquoting) is shown as a filled black circle; the corresponding fungal control sample was excluded from analysis due to low read counts and is therefore not shown. Temporal comparisons within each temperature group were performed using post hoc Tukey's HSD with FDR correction for: **(b)** bacterial richness (observed ASVs, 16S V4), **(c)** bacterial evenness (ASVs, 16S V4), **(d)** fungal richness (observed ASVs, ITS), and **(e)** fungal evenness (ASVs, ITS). Time points sharing the same letter are not significantly different; those with different letters represent significant changes (adjusted $p < 0.05$).

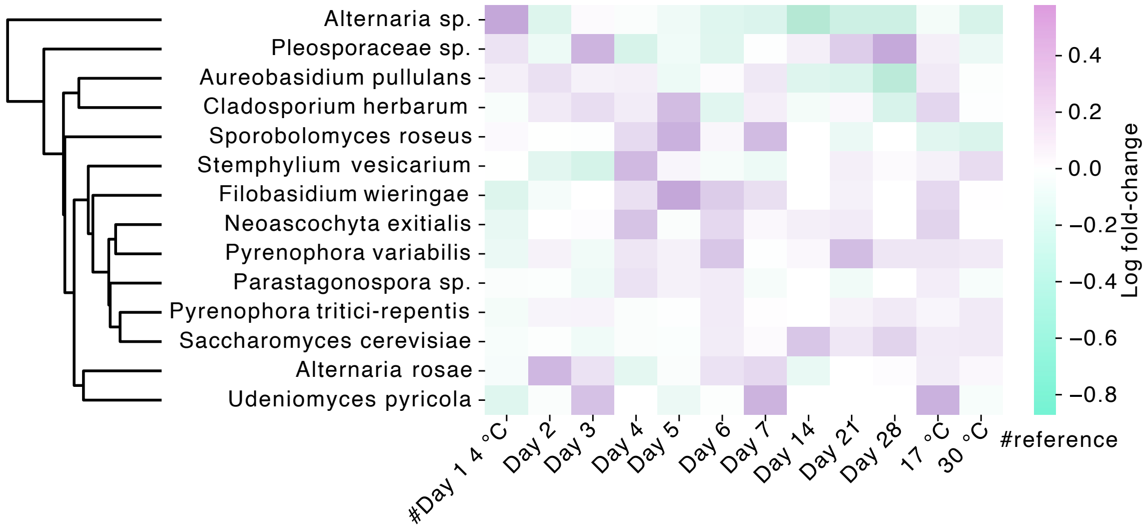

Supplementary Figure 6. Differential abundance analysis of fungal taxa in sourdough samples stored at 4 °C, 17 °C, and 30 °C over 28 days. Heatmap of log fold-changes in fungal (ITS) species relative to day 1 at 4 °C, computed using ANCOM-BC2. Taxa were hierarchically clustered on their log fold-changes using average linkage and Euclidean distance. No taxa exhibited statistically significant differential abundance across time or temperature (FDR-adjusted $q < 0.05$).

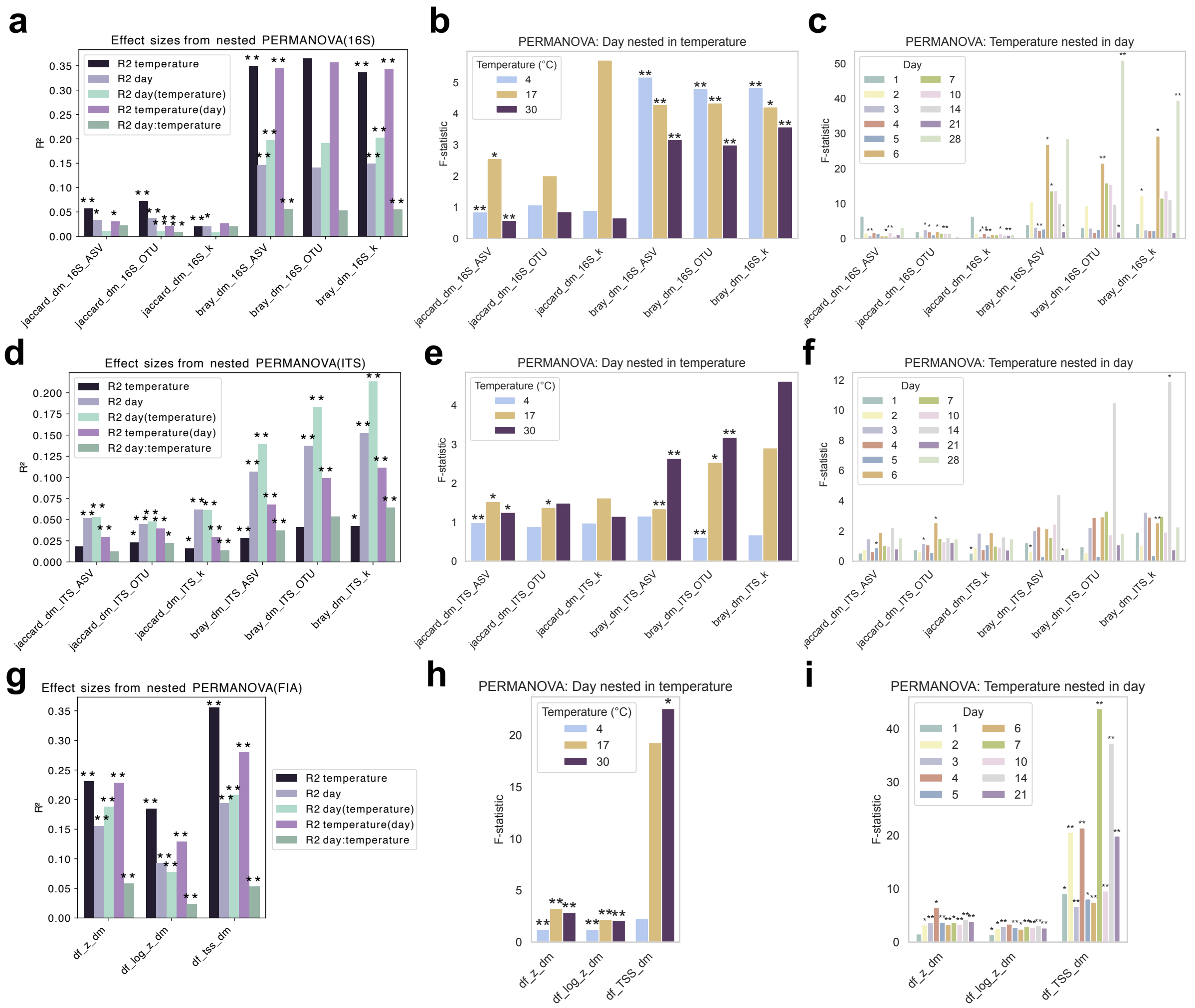

Supplementary Figure 7. Effect sizes of temperature, time, and their interactions on microbial community structure and metabolomic profiles in sourdough samples. Variance explained by each factor was assessed using PERMANOVA on distance matrices derived from bacterial and fungal community data (Jaccard and Bray–Curtis, using ASV, OTU, and k-mer feature tables) and from metabolomic profiles (FIA-MS, using z-score, log-z-score, or TSS-transformed data). **a,** Global effects of temperature, day, and their interaction on bacterial beta-diversity. **b,** Fine-grained effects of day within each temperature group on bacterial communities. **c,** Comparisons of temperatures within individual days for bacterial variance. **(d–f)** Equivalent analyses as in a–c, respectively, for fungal beta-diversity. **g,** Effects of temperature, day, and nested variables on global metabolome variance. **h,** Day effects nested within each temperature group, and **(i)** temperature effects nested within individual days for metabolomic variance. Statistical significance was assessed using 999 permutations (*p < 0.05, **p < 0.01, ***p < 0.001).

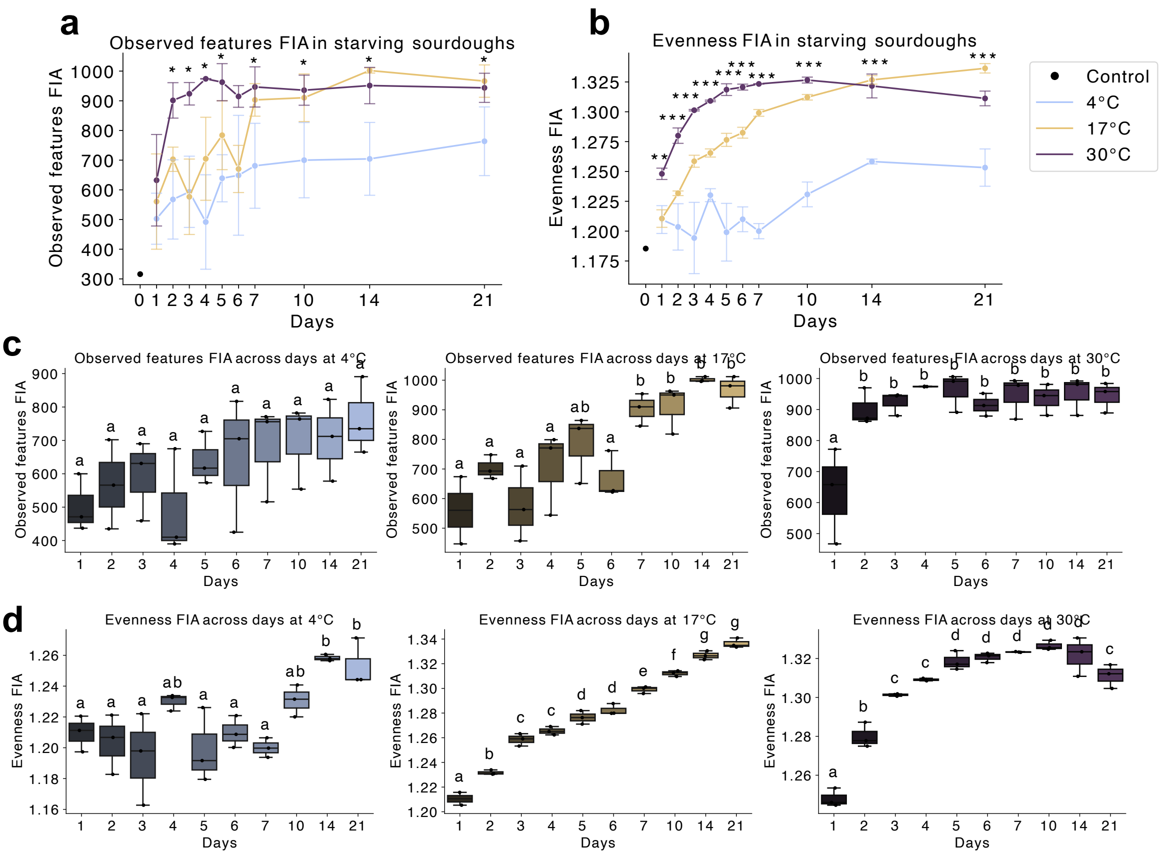

Supplementary Figure 8. Temporal dynamics of metabolite diversity in sourdough samples stored at 4 °C, 17 °C, and 30 °C over 21 days. a, Metabolic richness and **(b)** evenness, based on features detected by flow injection analysis–mass spectrometry (FIA-MS). Statistical comparisons across temperature groups at each time point were conducted using one-way ANOVA with FDR correction (*$p < 0.05$, **$p < 0.01$, ***$p < 0.001$). Baseline metabolite diversity from the control ('mother') sourdough at day 0 (upscaled overnight at 20 °C before aliquoting) is shown as a filled black circle. Within-group temporal comparisons were assessed using post hoc Tukey's HSD with FDR correction for: **(c)** observed metabolite features and **(d)** evenness. Time points sharing the same letter are not significantly different; those with different letters indicate statistically significant changes (adjusted $p < 0.05$).

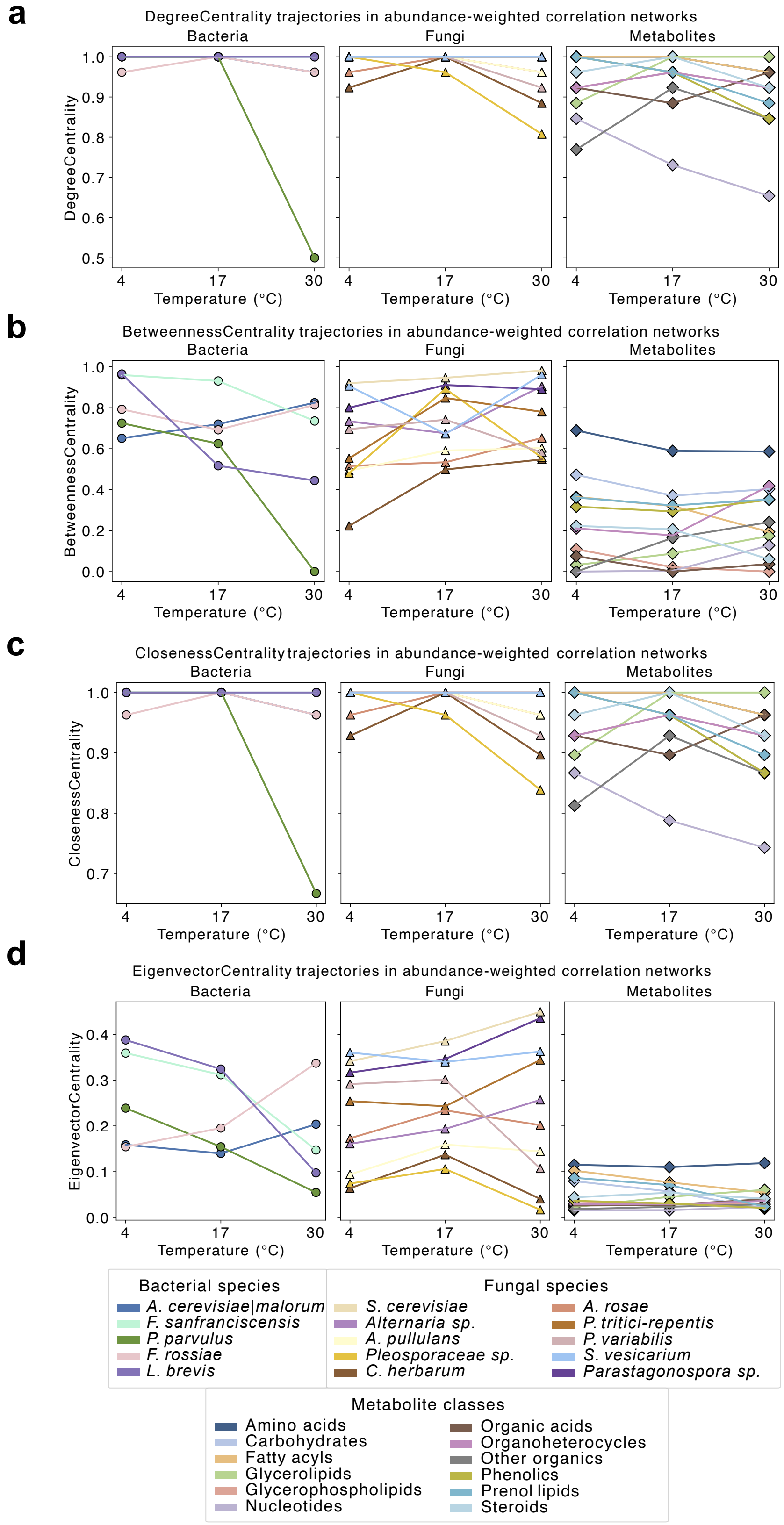

Supplementary Figure 9. Centrality metrics of abundance-weighted co-correlation networks in sourdough samples stored at 4 °C, 17 °C, and 30 °C. Network nodes represent bacterial taxa (left panels), fungal taxa (middle panels), and metabolite classes (right panels), with connections derived from correlation-based associations weighted by feature abundance. Centrality was assessed using four metrics: **(a)** degree centrality, **(b)** betweenness centrality,

(c) closeness centrality, and (d) eigenvector centrality. These metrics reflect the relative influence and connectivity of dominant and subdominant microbial and metabolite features across storage conditions.

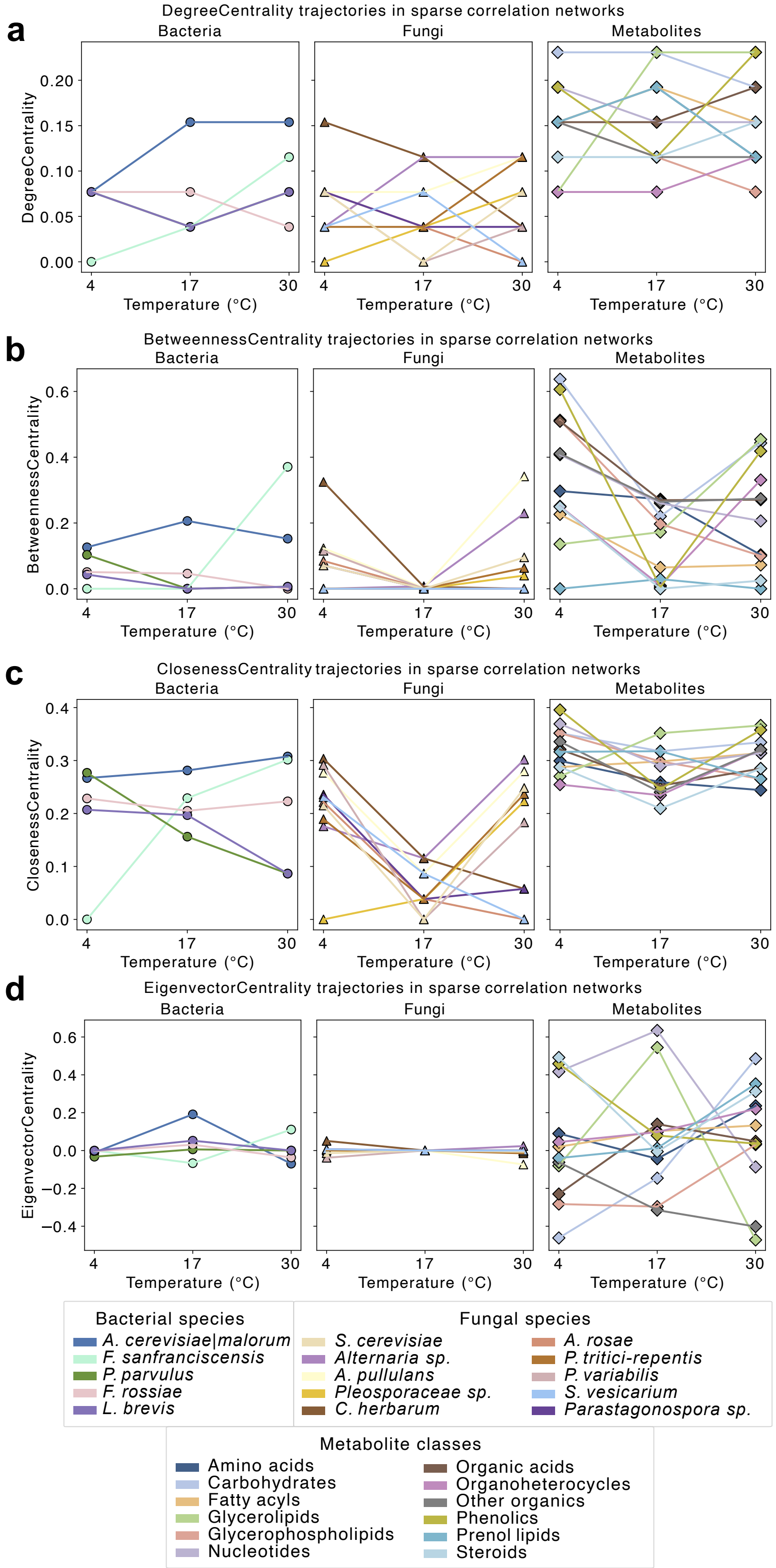

Supplementary Figure 10. Centrality metrics of sparse correlation networks in sourdough samples stored at 4 °C, 17 °C, and 30 °C. Network nodes represent bacterial

taxa (left panels), fungal taxa (middle panels), and metabolite classes (right panels), with edges derived from sparse inverse covariance estimates using the Graphical Lasso algorithm. Centrality was evaluated using: **(a)** degree centrality, **(b)** betweenness centrality, **(c)** closeness centrality, and **(d)** eigenvector centrality. These metrics characterize the structural connectivity and influence of microbial and metabolite features, independent of direct abundance co-correlations, across different storage conditions.

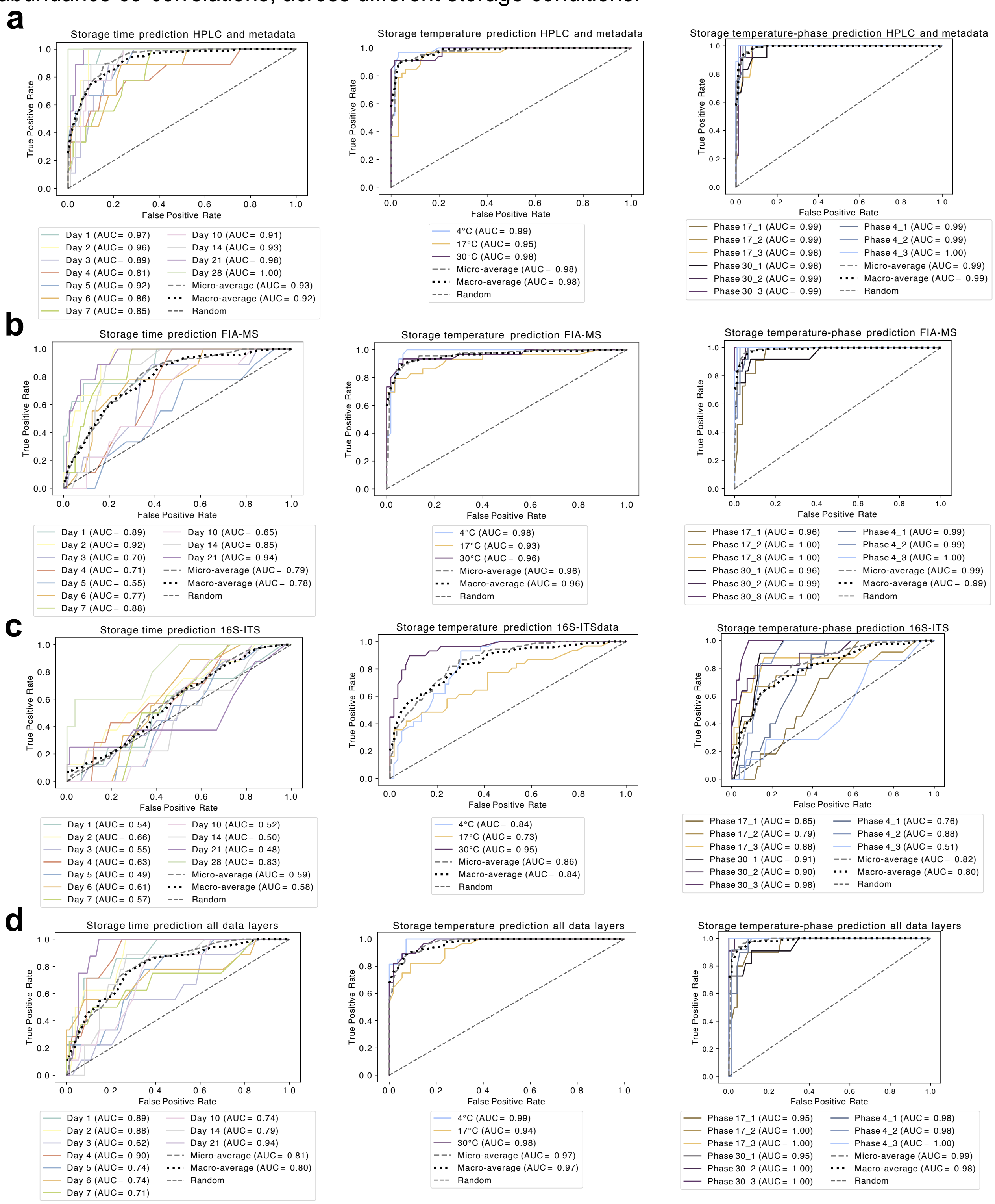

Supplementary Figure 11. Predictive accuracy of storage parameters based on multi-omics and physicochemical data layers. Classification performance of random forest models (100 estimators, 5-fold cross-validation) is reported as area under the curve (AUC) for

predicting storage day (left), storage temperature (middle), and combined storage phase (right; Phase 1: days 1–4, Phase 2: days 5–10, Phase 3: days 14–28). Models were trained using: **(a)** HPLC-derived sugar and acid profiles combined with colony-forming units (CFU), pH, and total titratable acidity (TTA); **(b)** metabolite features from FIA-MS; **(c)** CLR-transformed relative abundances from 16S V4 rRNA and ITS amplicon sequencing; and **(d)** a combined model integrating all features from (a), (b), and (c).

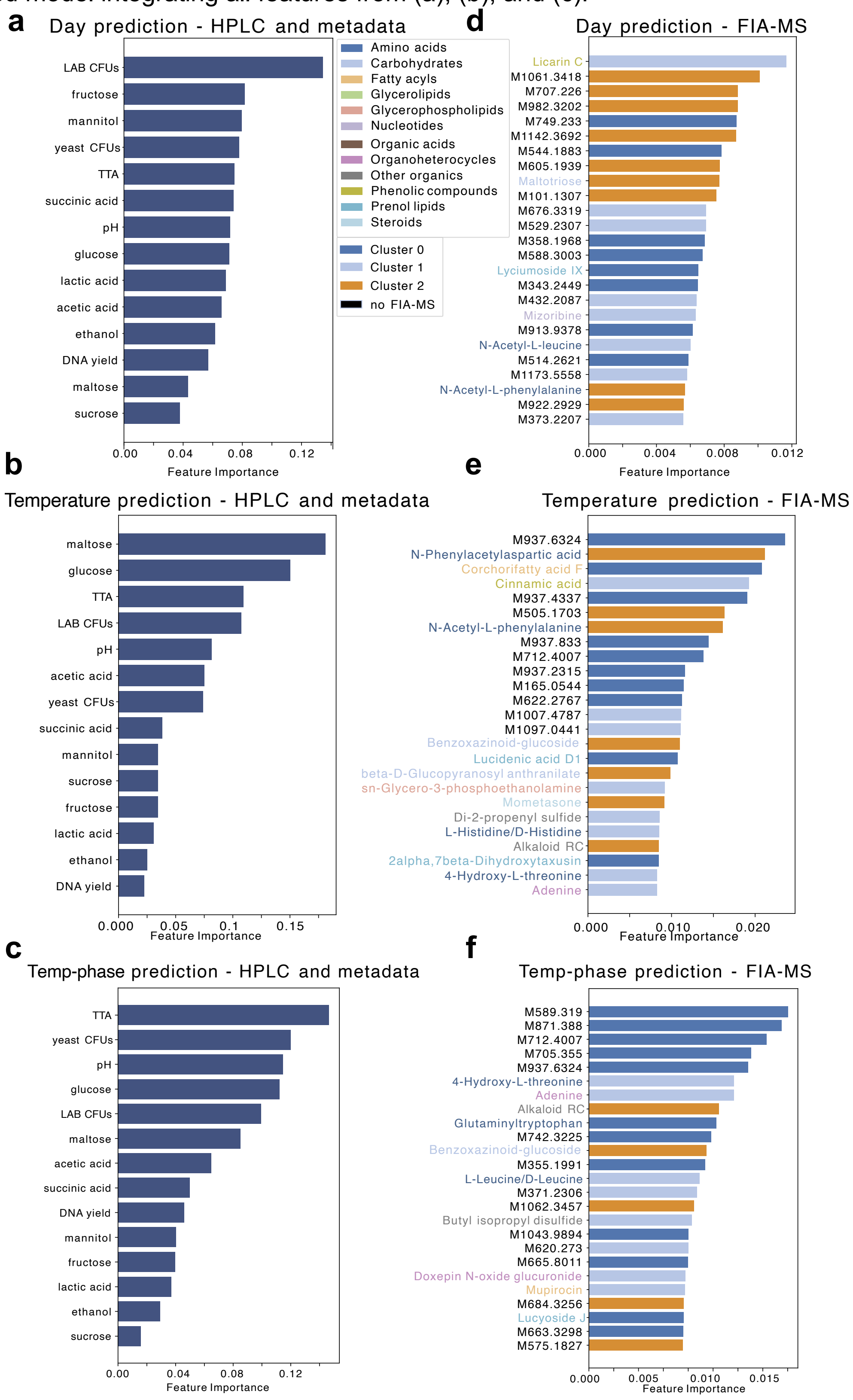

Supplementary Figure 12. Top predictive features for classifying storage parameters based on HPLC-metadata and FIA-MS data. Feature importance scores from random forest models are shown for predicting (top) storage day, (middle) storage temperature, and (bottom) combined storage phase (Phase 1: days 1–4, Phase 2: days 5–10, Phase 3: days 14–28), using: **(a–c)** HPLC-derived sugar and acid concentrations, CFU counts, pH, and total titratable acidity (TTA); and **(d–f)** metabolite features detected via FIA-MS. FIA-MS features were annotated where m/z values matched entries in the KEGG or HMDB databases within a mass tolerance of 0.002 Da, bars are colored according to the major dynamic metabolite clusters over time, and labels based on metabolite class. Models were trained with 100 estimators and 5-fold cross-validation. Top-ranked features reflect the most informative signals associated with temporal and temperature-driven changes in sourdough fermentation profiles.

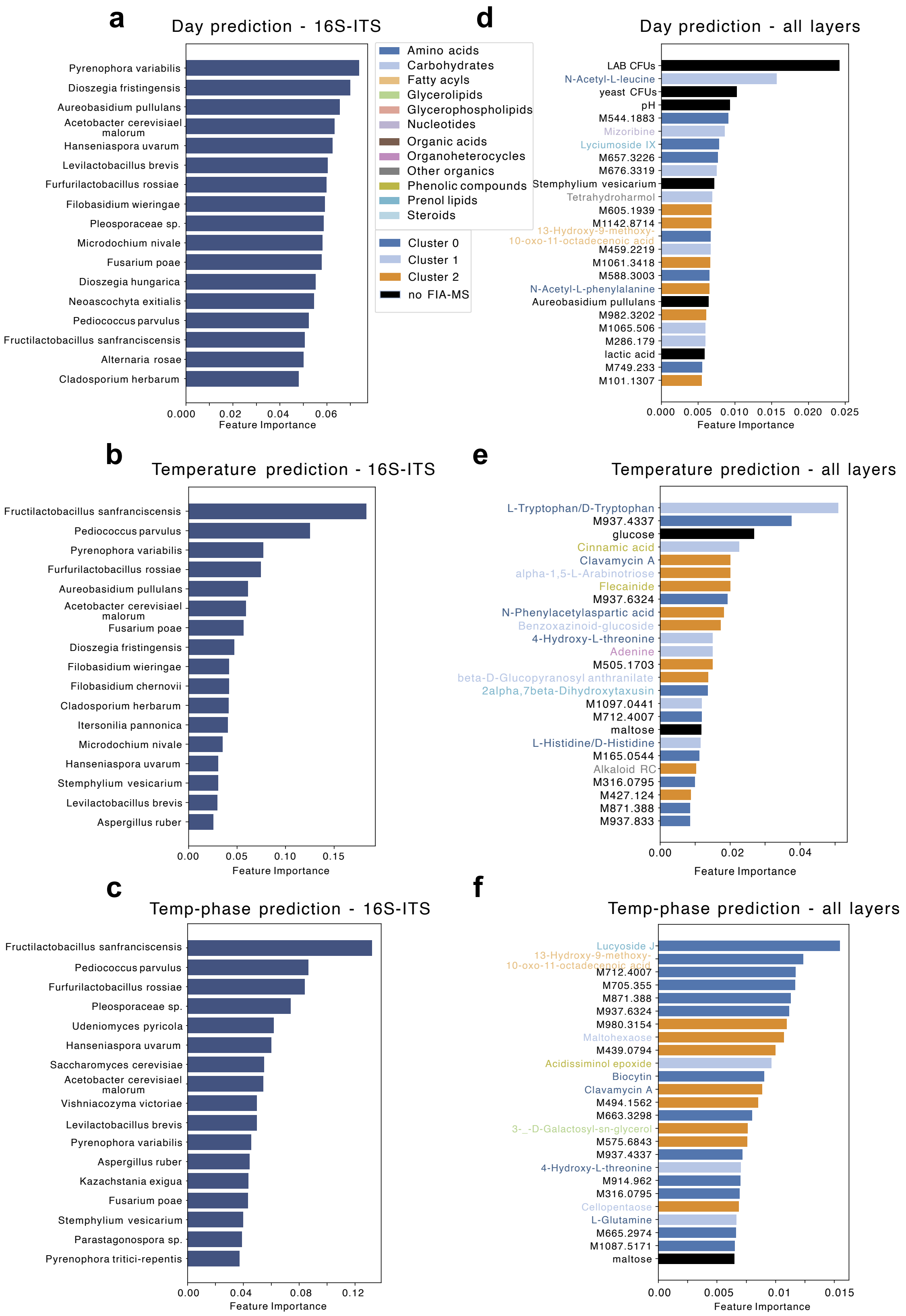

Supplementary Figure 13. Top predictive features for classifying storage parameters based on microbiome and fully integrated multi-omics data. Feature importance scores from random forest models are shown for predicting (top) storage day, (middle) storage temperature, and (bottom) combined storage phase per temperature (Phase 1: days 1–4, Phase 2: days 5–10, Phase 3: days 14–28), using: **(a–c)** microbiome features from combined bacterial (16S V4) and fungal (ITS) amplicon profiles; and **(d–f)** an integrated dataset

comprising microbiome, metabolome (FIA-MS and HPLC), CFU counts, pH, and total titratable acidity (TTA). Bars are colored according to the major dynamic FIA-MS metabolite clusters over time, and labels based on FIA-MS metabolite class. All models were trained with 100 estimators and 5-fold cross-validation. Top-ranked features represent the most informative variables contributing to the discrimination of temporal and temperature-dependent sourdough changes.

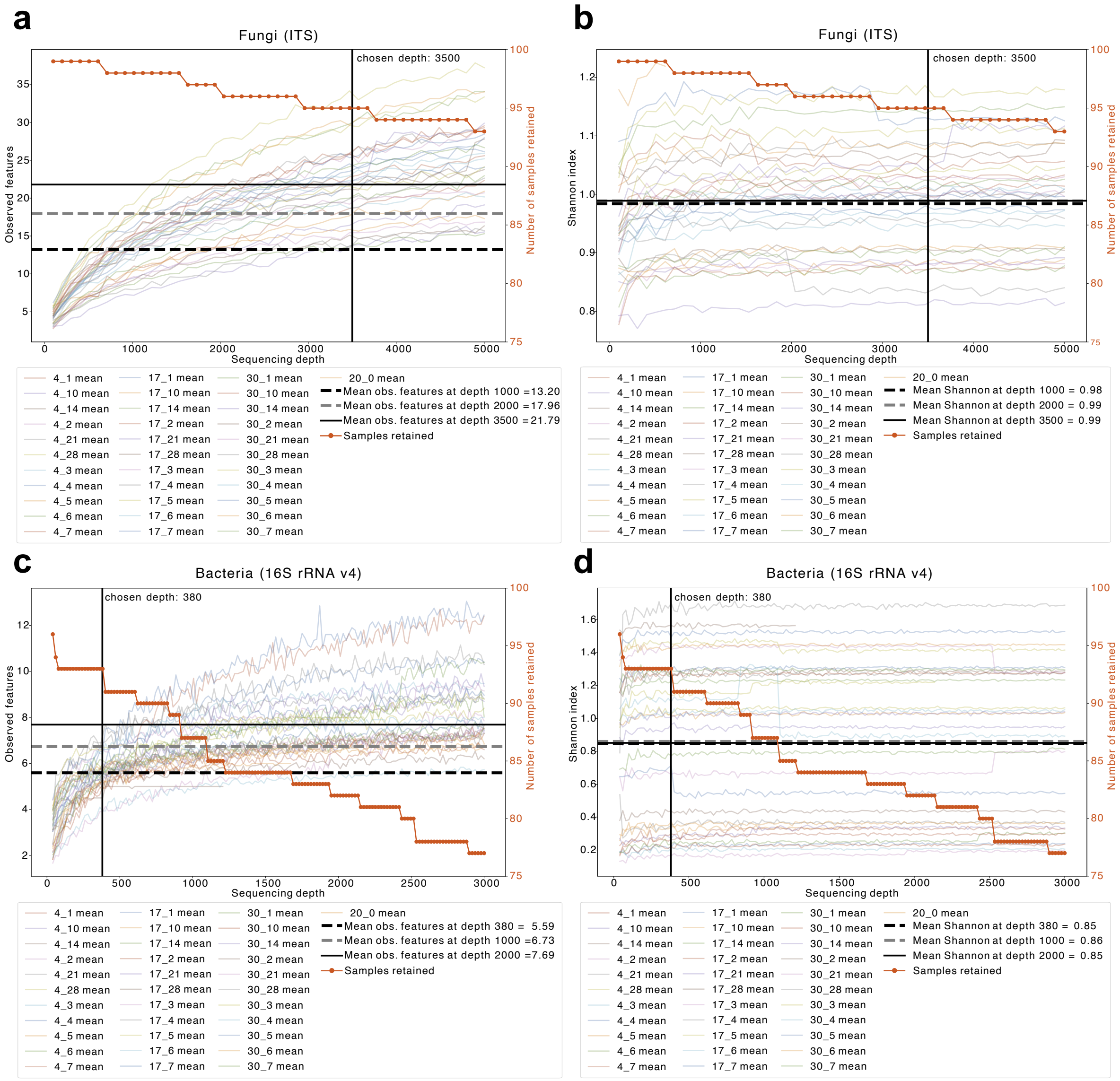

Supplementary Figure 14. Alpha-rarefaction curves and sample retention profiles for fungal and bacterial communities. (a, b) Fungal ASVs: rarefaction curves overlaid with sample retention curves for (a) observed features and (b) Shannon diversity; the selected sequencing depth of 3500 is indicated. **(c, d)** Bacterial ASVs: corresponding overlays for (c) observed features and (d) Shannon diversity, highlighting a chosen depth of 380 as the optimal trade-off between diversity representation and sample retention.

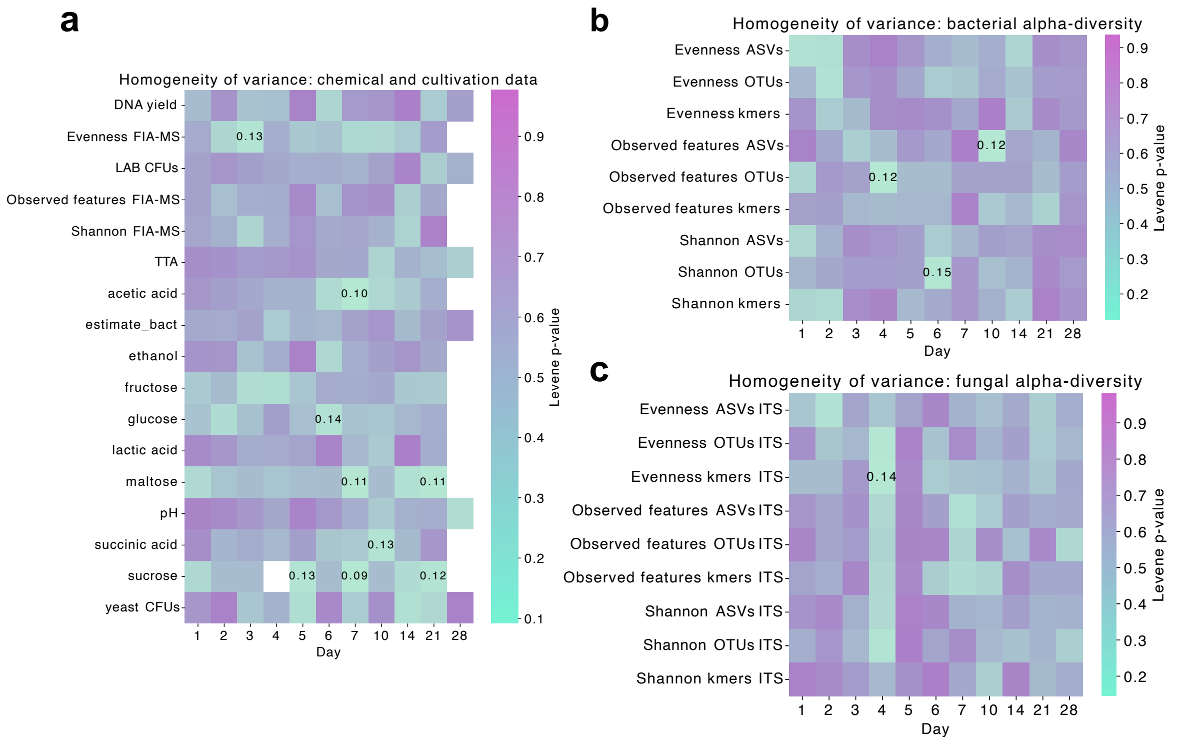

Supplementary Figure 15. Homogeneity of variance evaluation across temperatures. Heatmaps showing Levene's test p-values for comparing variance across storage temperatures (4 °C, 17 °C, and 30 °C) per day and metric. **a,** Chemical and cultivation-based data (HPLC, FIA-MS alpha-diversity, CFUs, DNA yield, and qPCR-based bacterial load estimate); **(b)**, bacterial alpha-diversity metrics; **(c)**, fungal alpha-diversity metrics across ASV, OTU, and k-mer resolutions. This analysis was conducted to evaluate the assumption of equal variances prior to performing ANOVA at each timepoint. P-values < 0.15 are annotated, yet none fall below the conventional threshold of 0.05, indicating no statistically significant heterogeneity of variance between temperature groups. These results support the suitability of ANOVA for downstream comparisons, even under small sample sizes. Chemical data (HPLC and FIA-MS) was not acquired for day 28 samples, therefore no p-values are displayed in (a) for those metrics at day 28.

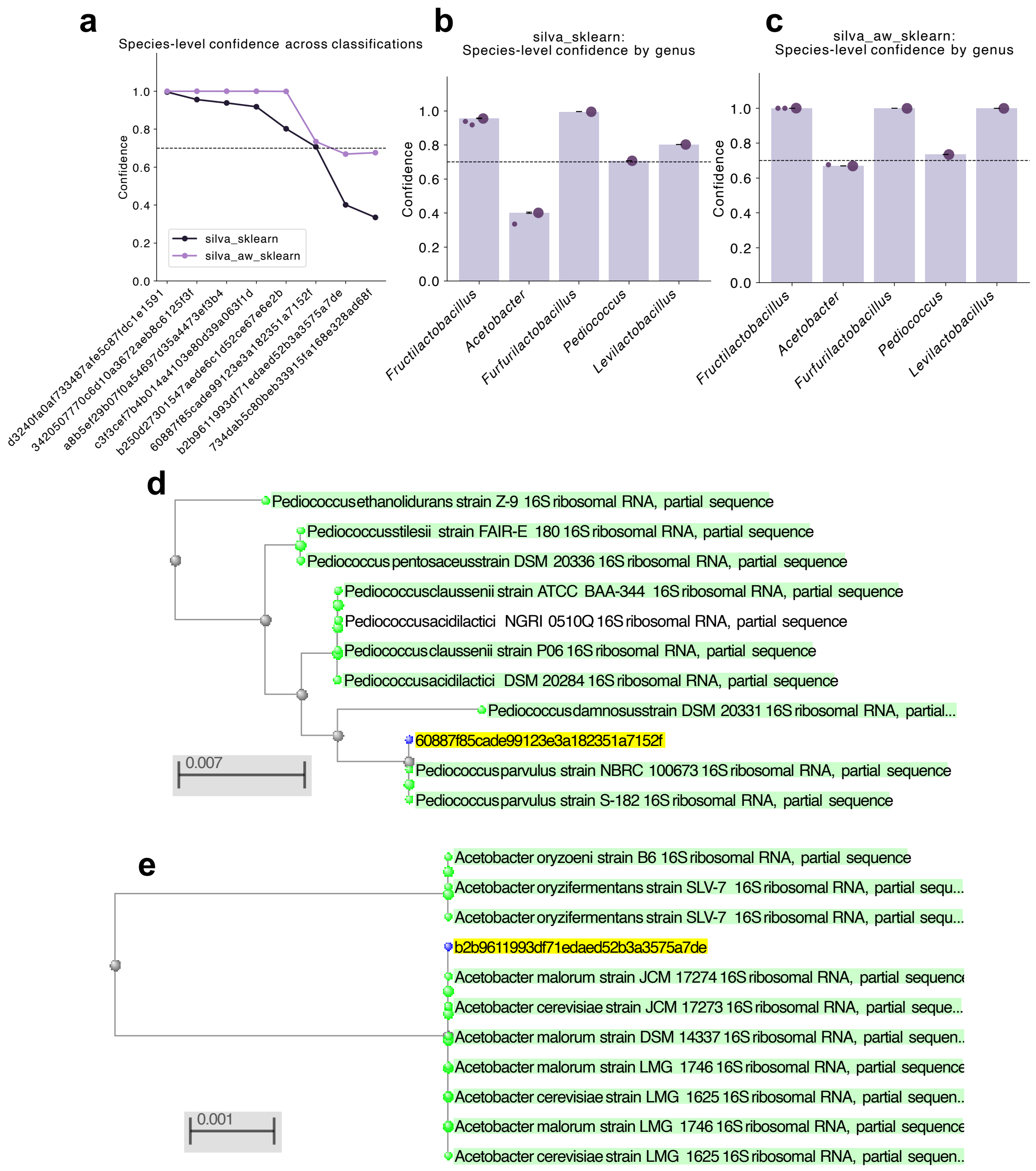

Supplementary Figure 16. Species-level classification probabilites for 16Sv4 ASVs. a, Species-level classification confidence for each ASV obtained with Naïve Bayes classifiers trained on the SILVA 138.2 reference database using *q2-feature-classifier*, either unweighted or average-weighted (aw; SILVA pre-trained classifier). **b,c,** Distribution of species-level classification confidence by genus for ASVs classified with the unweighted **(b)** and average-weighted **(c)** SILVA classifier. Bars indicate mean confidence per genus; points represent individual ASVs, with point size scaled by ASV relative abundance. The horizontal line at confidence = 0.7 denotes a commonly used threshold above which species-level labels are typically considered reliable. **d,** BLAST pairwise-alignment tree used to manually validate the *Pediococcus* species assignment: ASV 60887f85cade99123e3a182351a7152f clusters most closely with *Pediococcus parvulus* 16S rRNA sequences from the "16S ribosomal RNA (Bacteria and Archaea type strains)" database. **e,** BLAST pairwise-alignment tree used to manually validate *Acetobacter* species assignments: ASV b2b9611993df71edaed52b3a3575a7de clusters most closely with *Acetobacter malorum* and *Acetobacter cerevisiae* 16S rRNA sequences from the same database.

Supplementary tables

Supplementary Table 1. Composition and 16S rRNA gene copy number normalization of the ZymoBIOMICS Microbial Community DNA standard. This table lists microbial species, relative DNA abundance, genome size, ploidy, 16S/18S rRNA gene copy number, genome mass, and the calculated number of genomes and 16S genes per µL assuming 100 ng total DNA input. All values for genome size, ploidy, copy number, and genome mass were obtained from the ZymoBIOMICS Microbial Community DNA Standard (Cat. No. D6305) reference data. The total bacterial 16S rRNA gene abundance per µL with 100 ng total DNA was calculated as 1.58×10^{11} copies, corresponding to 2.65×10^{10} bacterial genomes. A mean normalization factor of 5.95 16S rRNA gene copies per genome can be derived by dividing total 16S abundance by total genome number ($1.58 \times 10^{11} / 2.65 \times 10^{10}$), a fixed factor of 5 was applied in this study, reflecting typical 16S copy numbers in dominant taxa such as the *Lactobacillus* representative in the standard, *Lactobacillus fermentum*. This correction factor was used to normalize 16S qPCR-based bacterial load estimates in sourdough samples, together with the assumption of 1.58×10^{9} gene copies per ng DNA.

Species	% DNA	ng DNA per 1 uL	genome size (Mb)	ploidy	16S/18S copy number	genome mass (in ng)	genomes per uL (100 ng)	16S genes per 1 uL with 100 ng DNA
Listeria monocytogenes	0.12	12	2992	1	6	3.23E-09	3715754833	22294529000
Pseudonomas aeruginosa	0.12	12	6792	1	4	7.33E-09	1636857842	6547431367
Bacillus subtilis	0.12	12	4045	1	10	4.37E-09	2748464391	27484643910
Escherichia coli	0.12	12	4875	1	7	5.26E-09	2280520710	15963644970
Salmonella enterica	0.12	12	4760	1	7	5.14E-09	2335617324	16349321267
Lactobacillus fermentum	0.12	12	1905	1	5	2.06E-09	5835978195	29179890975
Enterococcus faecalis	0.12	12	2845	1	4	3.07E-09	3907746384	15630985535
Staphylococcus aureus	0.12	12	2730	1	6	2.95E-09	4072358411	24434150465
Saccharomyces cerevisiae	0.02	2	12100	2	109	1.31E-08	153134138.6	16691621106
Cryptococcus neoformans	0.02	2	18900	2	60	2.04E-08	98038258.04	5882295482

Supplementary Table 2. Gradient used for Carbohydrate separation by HPLC.

Time (min.)	*Flow rate (mL/min.)*	*Mobile phase 1 in %*	*Mobile phase 2 in %*	*Mobile phase 3 in %*
0	0.25	35	0.2	64.8
5	0.25	35	0.2	64.8
20	0.25	35	3	62
30	0.25	60	30	10
35	0.25	35	0.2	64.8
45	0.25	35	0.2	64.8

Supplementary Table 3. Manual validation of *Pediococcus* ASV by BLAST against type-strain 16S rRNA sequences. Top five BLAST hits for ASV 60887f85cade99123e3a182351a7152f queried against the "16S ribosomal RNA (Bacteria and Archaea type strains)" database are shown.

Description	***Scientific Name***	***Max Score***	***Total Score***	***Query Cover***	***E value***	***Per. ident***	***Acc. Len***	***Accession***
Pediococcus parvulus strain NBRC 100673 16S ribosomal RNA, partial sequence	Pediococcus parvulus	468	468	100%	9E-132	100.00	1501	NR_113922.1
Pediococcus parvulus strain S-182 16S ribosomal RNA, partial sequence	Pediococcus parvulus	468	468	100%	9E-132	100.00	1436	NR_029136.1
Pediococcus claussenii strain ATCC BAA-344 16S ribosomal RNA, partial sequence	Pediococcus claussenii	457	457	100%	2E-128	99.21	1567	NR_075029.1
Pediococcus claussenii strain P06 16S ribosomal RNA, partial sequence	Pediococcus claussenii	457	457	100%	2E-128	99.21	1472	NR_042232.1
Pediococcus acidilactici DSM 20284 16S ribosomal RNA, partial sequence	Pediococcus acidilactici DSM 20284	457	457	100%	2E-128	99.21	1569	NR_042057.1

Supplementary Table 4. Manual validation of *Acetobacter* ASVs by BLAST against type-strain 16S rRNA sequences. Top five BLAST hits for ASV b2b9611993df71edaed52b3a3575a7de and 734dab5c80beb33915fa168e328ad68f queried against the "16S ribosomal RNA (Bacteria and Archaea type strains)" database are shown.

Acetobacter ASV1: b2b9611993df71edaed52b3a3575a7de

Description	*Scientific Name*	*Max Score*	*Total Score*	*Query Cover*	*E value*	*Per. ident*	*Acc. Len*	*Accession*
Acetobacter malorum strain JCM 17274 16S ribosomal RNA, partial sequence	Acetobacter malorum	468	468	100%	9E-132	100.00	1412	NR_113553.1
Acetobacter cerevisiae strain JCM 17273 16S ribosomal RNA, partial sequence	Acetobacter cerevisiae	468	468	100%	9E-132	100.00	1412	NR_113552.1
Acetobacter malorum strain DSM 14337 16S ribosomal RNA, partial sequence	Acetobacter malorum	468	468	100%	9E-132	100.00	1353	NR_118164.1
Acetobacter malorum strain LMG 1746 16S ribosomal RNA, partial sequence	Acetobacter malorum	468	468	100%	9E-132	100.00	1355	NR_118163.1
Acetobacter cerevisiae strain LMG 1625 16S ribosomal RNA, partial sequence	Acetobacter cerevisiae	468	468	100%	9E-132	100.00	1358	NR_118162.1

Acetobacter ASV2: 734dab5c80beb33915fa168e328ad68f

Description	*Scientific Name*	*Max Score*	*Total Score*	*Query Cover*	*E value*	*Per. ident*	*Acc. Len*	*Accession*
Acetobacter malorum strain JCM 17274 16S ribosomal RNA, partial sequence	Acetobacter malorum	462	462	100%	4E-130	99.60	1412	NR_113553.1
Acetobacter cerevisiae strain JCM 17273 16S ribosomal RNA, partial sequence	Acetobacter cerevisiae	462	462	100%	4E-130	99.60	1412	NR_113552.1
Acetobacter malorum strain DSM 14337 16S ribosomal RNA, partial sequence	Acetobacter malorum	462	462	100%	4E-130	99.60	1353	NR_118164.1
Acetobacter malorum strain LMG 1746 16S ribosomal RNA, partial sequence	Acetobacter malorum	462	462	100%	4E-130	99.60	1355	NR_118163.1
Acetobacter cerevisiae strain LMG 1625 16S ribosomal RNA, partial sequence	Acetobacter cerevisiae	462	462	100%	4E-130	99.60	1358	NR_118162.1

Supplementary Table 5. Differentially abundant bacterial taxa in the reference group. ANCOM-BC2 identified taxa enriched in sourdough samples stored at 4 °C on day 1. For log-ratio analyses over time (see Fig. 2e), only taxa with absolute log-fold changes (|logFC|) > 0.75 were retained. Based on the enrichments observed here, *Acetobacter cerevisiae|malorum* was selected as the denominator taxon, while *Fructilactobacillus sanfranciscensis* and *Furfurilactobacillus rossiae* were used as numerator taxa for all subsequent log-ratio calculations across time points.

taxon	*logFC*	*qval*	*pval*
Acetobacter cerevisiae|malorum	-1.6052	0.97919	0.493079
Fructilactobacillus sanfranciscensis	2.588816	0.97919	0.231429
Pediococcus parvulus	-0.06316	0.97919	0.97919
Furfurilactobacillus rossiae	0.94978	0.97919	0.666257
Levilactobacillus brevis	0.622505	0.97919	0.805273

Supplementary Table 6. Differentially abundant fungal taxa in the reference group. ANCOM-BC2 identified taxa enriched in sourdough samples stored at 4 °C on day 1. For log-ratio analyses over time (see Fig. 2f), only taxa with absolute log-fold changes (|logFC|) > 0.1 were retained. Based on the enrichments observed here, *Filobasidium wieringae, Neoascochyta exitialis, Pyrenophora tritici-repentis, Pyrenophora variabilis* and *Udeniomyces pyricola* were selected as the denominator taxa, while *Alternaria sp.* and *Pleosporaceae sp.* were used as numerator taxa for all subsequent log-ratio calculations across time points.

taxon	*logFC*	*qval*	*pval*
Alternaria rosae	-0.085088	0.997784	0.98185
Alternaria sp.	0.578727	0.997784	0.852397
Aureobasidium pullulans	0.093354	0.997784	0.977036
Cladosporium herbarum	-0.068014	0.997784	0.984249
Filobasidium wieringae	-0.349554	0.997784	0.943255
Neoascochyta exitialis	-0.230582	0.997784	0.961077
Parastagonospora sp.	-0.053159	0.997784	0.990194
Pleosporaceae sp.	0.179644	0.997784	0.9592
Pyrenophora tritici-repentis	-0.107098	0.997784	0.977447
Pyrenophora variabilis	-0.19592	0.997784	0.959906
Saccharomyces cerevisiae	-0.075105	0.997784	0.98019
Sporobolomyces roseus	0.036263	0.997784	0.994492
Stemphylium vesicarium	-0.011866	0.997784	0.997784
Udeniomyces pyricola	-0.325637	0.997784	0.956649

Supplementary Table 7. Concordance between bacterial community composition and metabolome profiles. Procrustes analysis comparing 16S v4 rRNA gene-based Bray–Curtis distances with various transformed and dimensionally reduced FIA-MS metabolomics datasets.

comparison	*Observed Disparity*	*95% CI*	*pval*
FIA_z_PCoA vs 16S_PCoA	0.591	[0.953632, 0.998986]	0.001
FIA_tss_PCoA vs 16S_PCoA	0.437	[0.946777, 0.999179]	0.001
FIA_tss_PCA vs 16S_PCoA	0.499	[0.944602, 0.998966]	0.001
FIA_z_PCA vs 16S_PCoA	0.591	[0.947394, 0.998983]	0.001

Supplementary Table 8. Temporal concordance between bacterial communities and metabolome profiles. Procrustes analysis comparing Bray–Curtis distances from 16S V4 rRNA gene data and TSS-normalized FIA-MS metabolomics profiles, both projected into PCoA space across time points.

day	*disparity*	*pval*
1	0.5755	0.0350
2	0.3473	0.0080
3	0.5785	0.0180
4	0.7841	0.3107
5	0.6087	0.0380
6	0.2776	0.0020
7	0.2416	0.0030
10	0.2974	0.0050
14	0.2552	0.0040
21	0.5397	0.0539

Supplementary Table 9. Temporal concordance by temperature between bacterial communities and metabolome profiles. Procrustes analysis comparing Bray–Curtis distances from 16S V4 rRNA gene data and TSS-normalized FIA-MS metabolomics profiles, both projected into PCoA space across time points per temperature.

day	*temperature*	*disparity*	*pval*
1	4	0.938	1.000
2	4	0.520	0.677
3	4	0.797	0.689
4	4	0.090	0.001
5	4	0.014	0.191
6	4	0.534	0.341
7	4	0.616	0.495
10	4	0.117	0.169
14	4	0.042	0.316
21	4	0.000	0.001
1	17	0.000	0.001
2	17	0.472	0.479
3	17	0.046	0.001
4	17	0.000	1.000
5	17	0.248	0.353
6	17	0.310	0.371
7	17	0.247	0.518
10	17	0.950	1.000
14	17	0.285	0.515
21	17	0.023	0.155
1	30	0.848	0.687
2	30	0.000	1.000
3	30	0.266	0.154
4	30	0.749	0.683
5	30	0.389	0.543
6	30	0.247	0.656
7	30	0.324	0.510
10	30	0.591	0.836
14	30	0.808	0.685
21	30	0.123	0.170
